# Early insulin combined with metformin therapy and long-term risks of cardiovascular disease, kidney disease, and cancer in newly diagnosed type 2 diabetes: a real-world cohort study in China

**DOI:** 10.3389/fendo.2026.1877839

**Published:** 2026-09-21

**Authors:** Luning Yan, Qingxin Luo, Juying Wang, Shuo Zhang, Ruize Xu, Mingyue Lv, Ping Su, Shuaijie Zhang, Xiaokang Ji, Gaopei Zhu, Fuzhong Xue

**Affiliations:** 1 Department of Medical Dataology, School of Public Health, Cheeloo College of Medicine, Shandong University, Jinan, China; 2 National Institute of Health and Medical Big Data, Jinan, China; 3 Qilu Hospital of Shandong University, Jinan, China; 4 School of Information Science and Engineering, Shandong Normal University, Jinan, Shandong, China; 5 National Administration of Health Data, Jinan, China; 6 Beijing Institute of Mathematical Sciences and Applications, Beijing, China; 7 Yau Mathematical Sciences Center, Tsinghua University, Beijing, China; 8 Shandong Mental Health Center, Jinan, China

**Keywords:** active-comparator new-user design, early insulin combined with metformin therapy, L1-regularized propensity score method, longitudinal association, real-world study, type 2 diabetes mellitus

## Abstract

**Objective:**

To evaluate the longitudinal associations of early insulin combined with metformin therapy, compared with metformin monotherapy, with subsequent risks of stroke, heart failure, chronic kidney disease, and cancer among adults with newly diagnosed type 2 diabetes using real-world data.

**Methods:**

We conducted an active-comparator new-user cohort study using the Cheeloo LEAD electronic health record database (Shandong, China; 2014–2021). Adults aged 40–85 years who initiated early insulin combined with metformin therapy or metformin monotherapy during 2014–2017 were included in the common parent cohort (n=19,908; 4,304 [21.6%] and 15,604 [78.4%], respectively). Follow-up began at each treatment strategy initiation date (t0). Four independent outcome-specific subcohorts excluded patients with the corresponding pre-existing outcome and those with the outcome recorded within 30 days after t0. The combination group required at least 14 days of overlapping insulin and metformin use. L1-regularized propensity score matching and Cox proportional hazards models were used to estimate hazard ratios (HRs) and 95% confidence intervals (CIs) over up to 1,500 days. Sensitivity analyses used a 180-day outcome-exclusion window.

**Results:**

Compared with metformin monotherapy, early insulin combined with metformin therapy was associated with a lower hazard of stroke (HR 0.88, 95% CI 0.78–0.99) and higher hazards of heart failure (HR 1.18, 95% CI 1.02–1.36), chronic kidney disease (HR 1.37, 95% CI 1.03–1.83), and cancer (HR 1.37, 95% CI 1.14–1.66). Exploratory subgroup estimates varied by age and sex; no formal interaction tests were performed. Results were broadly consistent in sensitivity analyses.

**Conclusion:**

Early insulin combined with metformin therapy and metformin monotherapy showed different longitudinal associations with the four outcomes. These findings should be interpreted in light of residual confounding by baseline disease severity and treatment indication and may inform risk monitoring in routine care.

## 1 Introduction

Diabetes mellitus has become a major global public health concern, with the prevalence of type 2 diabetes mellitus (T2DM) in China showing a steadily increasing trend (1). The prevalence of diabetes among Chinese adults aged 18 and above rose from 0.67% in 1980 to 11.2% in 2017 (2), and this upward trajectory has not yet reached a plateau. According to the Chinese guideline for the prevention and treatment of type 2 diabetes, metformin is recommended as the preferred initial pharmacological therapy for most patients, whereas early insulin therapy may be considered for newly diagnosed patients with marked hyperglycemia or related symptoms (3). T2DM accounts for over 90% of all diabetes cases in China (4). Without effective therapeutic control, patients with T2DM are at high risk for developing serious complications, such as cardiovascular disease, nephropathy, and neuropathy, which can severely compromise quality of life (5, 6).

With advances in the understanding of T2DM pathophysiology and the development of standardized treatment protocols, a stepwise therapeutic approach has been established—beginning with metformin as first-line therapy, followed by the sequential addition of second- and third-line agents (3). In parallel, early insulin combined with metformin therapy has been recommended for patients with poor glycemic control or those in the early stages of the disease (7), aiming to reduce the risk of early complications (8, 9) and adverse cardiovascular outcomes (10). Active-comparator new-user designs compare clinically relevant treatment alternatives and can reduce confounding related to treatment indication and healthcare-seeking behavior (11). Metformin monotherapy was selected as the active comparator because it was the guideline-recommended initial pharmacological treatment for most patients with newly diagnosed type 2 diabetes who required drug therapy in China during the study period (3). Thus, it represented a clinically meaningful alternative to Early insulin combined with metformin therapy. Nevertheless, the long-term safety and generalizability of early insulin combined with metformin therapy remain underexplored in China. This is partly due to its recent inclusion in national guidelines and various complexities in clinical practice, including limited provider knowledge, time constraints, and patient reluctance (12, 13). To address these gaps, the present study leverages population-based real-world data to provide additional evidence supporting rational and safe treatment strategies.

This study utilized data from the Cheeloo LEAD database (14) to implement a new-user design research framework (15, 16) to evaluate longitudinal associations between early insulin combined with metformin therapy, compared with metformin monotherapy, and long-term outcomes in patients with T2DM. Exploratory subgroup analyses were performed by age and sex to describe subgroup-specific estimates; no formal interaction tests were performed. In addition, sensitivity analyses using varying post-t0 outcome-exclusion windows were conducted to ensure the robustness of our findings. These results are intended to supplement existing evidence and inform optimized, individualized therapeutic strategies for T2DM management.

## 2 Materials and methods

### 2.1 Data sources

This study utilized the Cheeloo Lifespan Electronic Health Research Data-library (Cheeloo LEAD), a longitudinal electronic health record (EHR) database derived across 5,152,597 individuals selected through cluster random sampling from 39 counties or districts in Shandong Province. This sample represents a source population of approximately 101 million residents across 136 counties or districts. The database integrates and links 149 data tables via unique personal identification numbers covering the complete life-course health information of residents from January 1, 2009, to the present. Data linkage across these tables was performed deterministically using a unique, encrypted personal identification number for each resident. In contrast to electronic medical records (EMRs), which primarily comprise digitized clinical documentation generated within an individual healthcare institution, EHRs integrate longitudinal health information across healthcare settings. The integrated sources include clinical encounter records, basic public health services, health examination records, disease surveillance, medical insurance claims, cause-of-death monitoring, and environmental and ecological data (14, 17–19). After applying inclusion and exclusion criteria, a total of 19,908 newly diagnosed type 2 diabetes mellitus patients aged between 40 and 85 years were identified from January 1, 2014, to December 31, 2017. Initial disease codes and Chinese diagnostic terms were standardized to three-character codes based on the 10th revision of the International Classification of Diseases (ICD-10), and the diagnostic data were harmonized accordingly.

### 2.2 Ethics statement

This study was approved by the Ethics Committee of the School of Public Health, Shandong University (Approval No. LL20240308). The requirement for informed consent was waived due to the retrospective nature of the study and the use of anonymized data.

### 2.3 Cohort design

Well-utilized real-world evidence can offer critical insights for evaluating the effectiveness and safety of medical interventions, thereby supporting the improvements of care and treatment strategies. However, methodological limitations may lead to biased conclusions, such as those arising from prevalent user bias (20). To address this concern, the present study adopted an active-comparator new-user design (11), in which the initiation of treatment was aligned with the start of follow-up, and potential confounders were defined and adjusted for as covariates. The design was specified using target-trial principles to align eligibility, treatment-strategy initiation, and follow-up (21). This approach was used to estimate longitudinal associations between the two treatment strategies and subsequent outcomes.

Participants entered the cohort at different calendar times and therefore had variable potential follow-up durations before the common study end date. Participants were followed until the earliest occurrence of the outcome of interest, death, 1,500 days after t0, or 31 December 2021. Participants who died before the outcome of interest were right-censored on the date of death, whereas those who remained event free were administratively right-censored at 1,500 days or 31 December 2021, whichever occurred first. Cox proportional hazards models were used to estimate longitudinal associations while accounting for right-censored person-time.

Cohort entry was restricted to treatment initiation on or before 31 December 2017, with a common study end date of 31 December 2021. Follow-up was capped at 1,500 days for all participants. The new-user design aligned treatment-strategy initiation, cohort entry, and follow-up at the strategy-specific t0.

As shown in **Figure 1**, this active-comparator new-user cohort used longitudinal EHR data from Shandong Province, China. Patients newly diagnosed with type 2 diabetes mellitus (ICD-10 code E11.900) between 1 January 2014 and 31 December 2017 were eligible if they initiated either Early insulin combined with metformin therapy or Metformin monotherapy. This study used a fixed baseline treatment-strategy classification rather than dynamic reclassification according to subsequent treatment changes. For the Early insulin combined with metformin therapy group, t0 was the date of the first insulin prescription in the combination strategy; for the Metformin monotherapy group, t0 was the date of the first metformin prescription. Follow-up began at the respective t0, and treatment classification was not subsequently updated. The Early insulin combined with metformin therapy group was required to have concurrent metformin use at t0 and at least 14 days of overlapping insulin and metformin exposure to confirm sustained combination therapy and exclude transient insulin use related to surgery, acute stress, or other short-term clinical circumstances. Patients receiving insulin without concurrent metformin were excluded. For the primary analysis, patients with the corresponding outcome recorded during the first 30 days after t0 were excluded from each outcome-specific subcohort. This rule was intended to reduce the influence of outcomes occurring after only a short period of treatment exposure and to minimize temporal ambiguity when treatment initiation and outcome diagnosis were recorded during the same period or hospitalization. For example, patients with heart failure recorded during the first 30 days after t0 were excluded from the heart failure subcohort; the same rule was applied to the stroke, CKD, and cancer subcohorts. This rule did not change the start of follow-up at t0. In the sensitivity analysis, the outcome-exclusion window was extended from 30 to 180 days while retaining the same t0 and follow-up definition.

**Figure 1:**
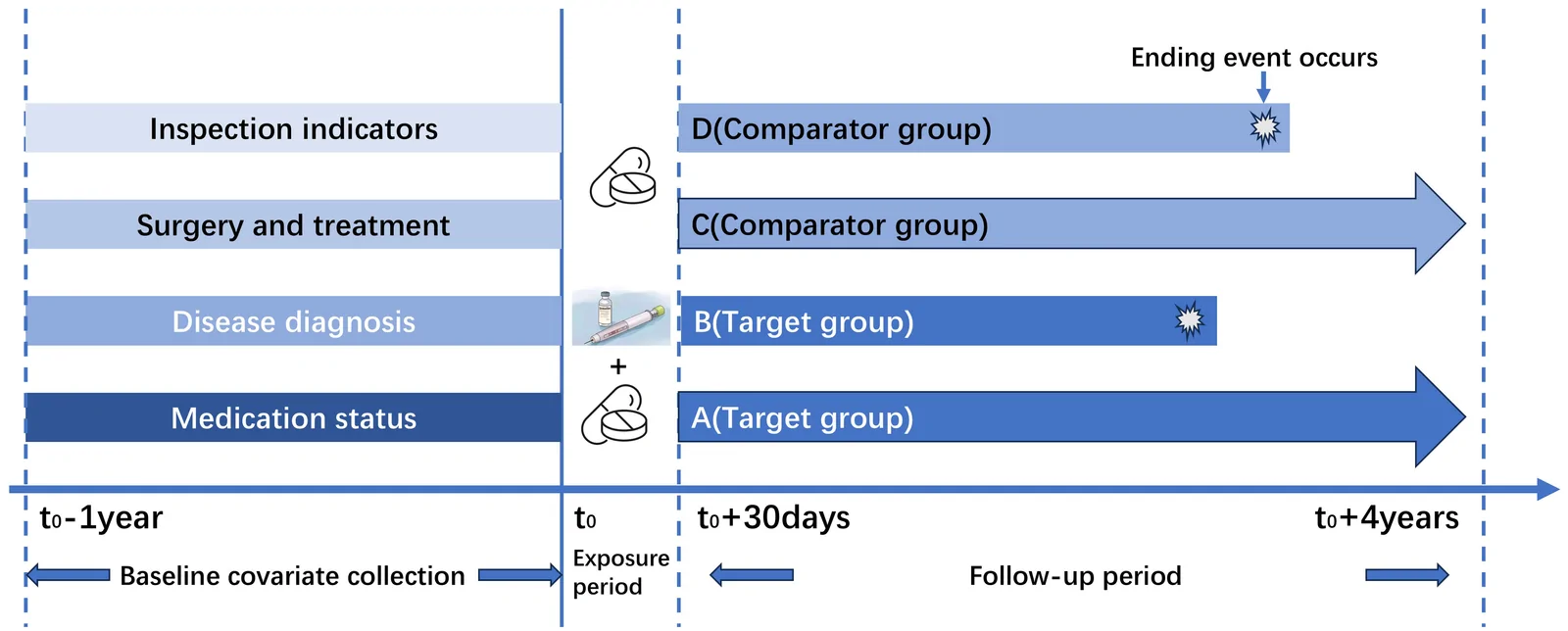
Study design and time alignment for the active-comparator new-user cohort. Baseline covariates were assessed during the year before the strategy-specific treatment initiation date (t0). Follow-up began at t0. For each outcome-specific subcohort, patients with the corresponding outcome recorded within 30 days after t0 were excluded; this exclusion window did not redefine t0. Early insulin combined with metformin therapy required concurrent metformin use at t0 and at least 14 days of overlapping exposure. Follow-up ended at the earliest of the outcome, death, 1,500 days after t0, or 31 December 2021. In sensitivity analyses, the outcome-exclusion window was extended to 180 days while retaining the same t0 and follow-up definition.

The following exclusion criteria were applied:

(1) patients with invalid or erroneous identification numbers; (2) patients aged <40 or >85 years at baseline; (3) patients with abnormal or missing sex records; (4) patients with major pancreatic diseases; and (5) patients whose first recorded initiation of glucose-lowering pharmacotherapy occurred outside the prespecified 2014–2017 cohort-entry period. Dialysis and kidney transplantation were additionally excluded from the CKD-specific subcohort.

Overall, cohorts were constructed in two steps. First, a common parent cohort was assembled using the uniform medication eligibility rules and general exclusion criteria. Second, four independent outcome-specific analytic subcohorts were constructed. The stroke subcohort excluded patients with a recorded history of stroke before t0; the heart failure subcohort excluded those with a recorded history of heart failure before t0; the CKD subcohort excluded those with pre-existing CKD, dialysis, or kidney transplantation before t0; and the cancer subcohort excluded those with any recorded malignant neoplasm before t0. Patients with major pancreatic disease were excluded from all four subcohorts. Each Cox model assessed the first recorded occurrence of the corresponding outcome after t0.

Follow-up began at the respective treatment-strategy initiation date (t0). Patients with the corresponding outcome recorded during the 30 days after t0 were excluded from each outcome-specific analysis. For each subcohort, participants were followed until the earliest occurrence of the outcome of interest, death, 1,500 days after t0, or 31 December 2021. Participants who died before the outcome of interest were right-censored on the date of death.

### 2.4 Exposure

This study focused on high-incidence complications of T2DM as follow-up outcomes (22), to evaluate longitudinal associations between early insulin combined with metformin therapy and subsequent outcomes. The Early insulin combined with metformin therapy group comprised patients who initiated insulin with concurrent metformin, whereas the Metformin monotherapy group comprised patients who initiated metformin without insulin (3). For the Early insulin combined with metformin therapy group, t0 was the date of the first insulin prescription in the combination strategy; for the Metformin monotherapy group, t0 was the date of the first metformin prescription. Follow-up began at the respective t0.

Early insulin combined with metformin therapy was defined with reference to the early-insulin criteria proposed by Luo et al. (10). Concurrent metformin use at t0 and at least 14 days of overlapping insulin and metformin exposure were required to confirm sustained combination therapy and exclude transient insulin use related to surgery, acute stress, or other short-term clinical circumstances. Patients receiving insulin without concurrent metformin were excluded (23).

### 2.5 Outcome definition

The primary outcomes included major long-term complications commonly observed in T2DM populations: Stroke (ICD-10 codes I60–I64, excluding I63.6), Heart failure (ICD-10 codes I50), Chronic kidney disease (CKD) (ICD-10 code N18), Cancer (ICD-10 codes C00–C97) (3).

Outcome ascertainment was based on ICD-10 diagnosis codes and recorded dates. Only the presence or absence of each outcome was captured; standardized clinical severity stages were not uniformly available for stroke, heart failure, CKD, or cancer, and outcome-specific severity stratification was not performed. For exploratory stroke subtype analyses, stroke events were classified as ischemic or hemorrhagic stroke according to ICD-10 diagnosis codes.

### 2.6 Covariate acquisition

Baseline covariates were ascertained during the one-year period before t0. The initial candidate covariate pool comprised demographic characteristics, pre-existing diseases, prior medication use, clinically relevant surgical procedures, diagnostic procedures, and other healthcare interventions. Surgical procedures were defined as therapeutic or operative interventions, whereas diagnostic procedures were defined as investigations performed to establish or evaluate clinical conditions. Dialysis was treated as an eligibility exclusion for the CKD subcohort rather than as a propensity score covariate. No laboratory or anthropometric measure of baseline disease severity, including HbA1c, fasting blood glucose, BMI, eGFR, urinary protein, or urine albumin, was incorporated into the final analytic dataset or the primary propensity score model because these data were substantially incomplete and were recorded at non-uniform time points during the baseline period.

Consistent with the Chinese guideline, short-term insulin treatment may be considered for newly diagnosed patients with HbA1c ≥9.0% or fasting plasma glucose ≥11.1 mmol/L who have marked hyperglycemic symptoms. This guideline-informed clinical context was considered when defining the treatment strategies but did not replace quantitative adjustment for laboratory measures (3).

### 2.7 Confounder control and matching

During the one-year baseline period before t0, we systematically extracted all recorded information within the prespecified covariate categories of demographic characteristics, pre-existing diseases, prior medication use, surgical procedures, and diagnostic procedures. Here, “all available covariates” referred to all recorded variables within these prespecified categories rather than every field in the database, and did not imply that every extracted variable entered the final propensity score model. The extracted variables were assembled into the initial candidate covariate pool. To improve comparability with respect to measured confounders, we applied L1-regularized propensity score (PS) matching (23, 24). Propensity score matching was used to improve balance in the distribution of observed baseline covariates between the treatment groups (25).

Owing to the high dimensionality of covariates available in the database, we constructed the PS model using an L1-penalized logistic regression approach, enabling covariate selection in a high-dimensional setting. The quality of the resulting pseudo-randomization was assessed using three diagnostic strategies: ① Evaluation of overlap and preference score distribution; ② Assessment of standardized mean differences (SMDs) to evaluate covariate balance; ③ Plotting Kaplan-Meier survival curves after matching to describe event-free survival in the two treatment groups (24).

For each outcome-specific analytic subcohort, variables with extremely low baseline prevalence were first removed during preliminary frequency screening. L1-regularized logistic regression was then applied to the remaining candidate covariates, and only variables with non-zero coefficients associated with treatment-group assignment were retained for propensity score estimation and matching. The variables retained in the propensity score model, including retained surgical and diagnostic procedure variables, and their balance before and after matching are reported in the existing **Supplementary Material**. Not all extracted variables entered the final propensity score model.

### 2.8 Statistical analysis

We summarized the number of individuals, incident cases, median follow-up time, and incidence density by sex and age group to describe outcome patterns across demographic subgroups. In the matched cohort created using L1-regularized propensity score matching, Kaplan–Meier (KM) curves were plotted to compare event-free survival between treatment groups (25). Covariate balance after matching was assessed using standardized mean differences (SMDs) and propensity score overlap diagnostics (reported in the **Supplementary Material**). Because matching achieved good balance on measured baseline covariates, the primary association estimate was obtained from a treatment-only Cox proportional hazards model in the matched cohort, using time since treatment initiation as the underlying time scale (26). Hazard ratios (HRs) and corresponding 95% confidence intervals (CIs) were calculated; results were considered statistically significant if the CI did not include 1.0.

Exploratory subgroup analyses were conducted by age (<60 vs. ≥60 years) and sex to describe subgroup-specific estimates. Formal interaction terms and P values for interaction were not prespecified or calculated; therefore, numerical variation or differences in statistical significance were not interpreted as evidence of subgroup heterogeneity. A sensitivity analysis extended the post-t0 outcome-exclusion window from 30 to 180 days while retaining the same strategy-specific t0 and follow-up definition.

All statistical analyses were conducted using R version 4.1.2 (27). Multiple R packages were utilized to facilitate data processing, modeling, and visualization.

### 2.9 Patient and public involvement

It was not appropriate or possible to involve patients or the public in the design, or conduct, or reporting, or dissemination plans of our research, as this was a retrospective cohort study utilizing anonymized electronic health record data from the Cheeloo LEAD database. No specific patient groups were consulted regarding the research question or outcome measures. Results will be disseminated to the relevant clinical communities through publication and conference presentations; however, direct dissemination to the individual study participants is not feasible due to the anonymized nature of the dataset.

## 3 Results

### 3.1 Cohort

As shown in **Figure 2**, 19,908 patients with newly diagnosed T2DM met the common parent-cohort criteria. Of these, 9,831 (49.4%) were male and 10,077 (50.6%) were female. A total of 15,604 patients (78.4%) initiated metformin monotherapy, and 4,304 (21.6%) initiated early insulin combined with metformin therapy.

**Figure 2:**
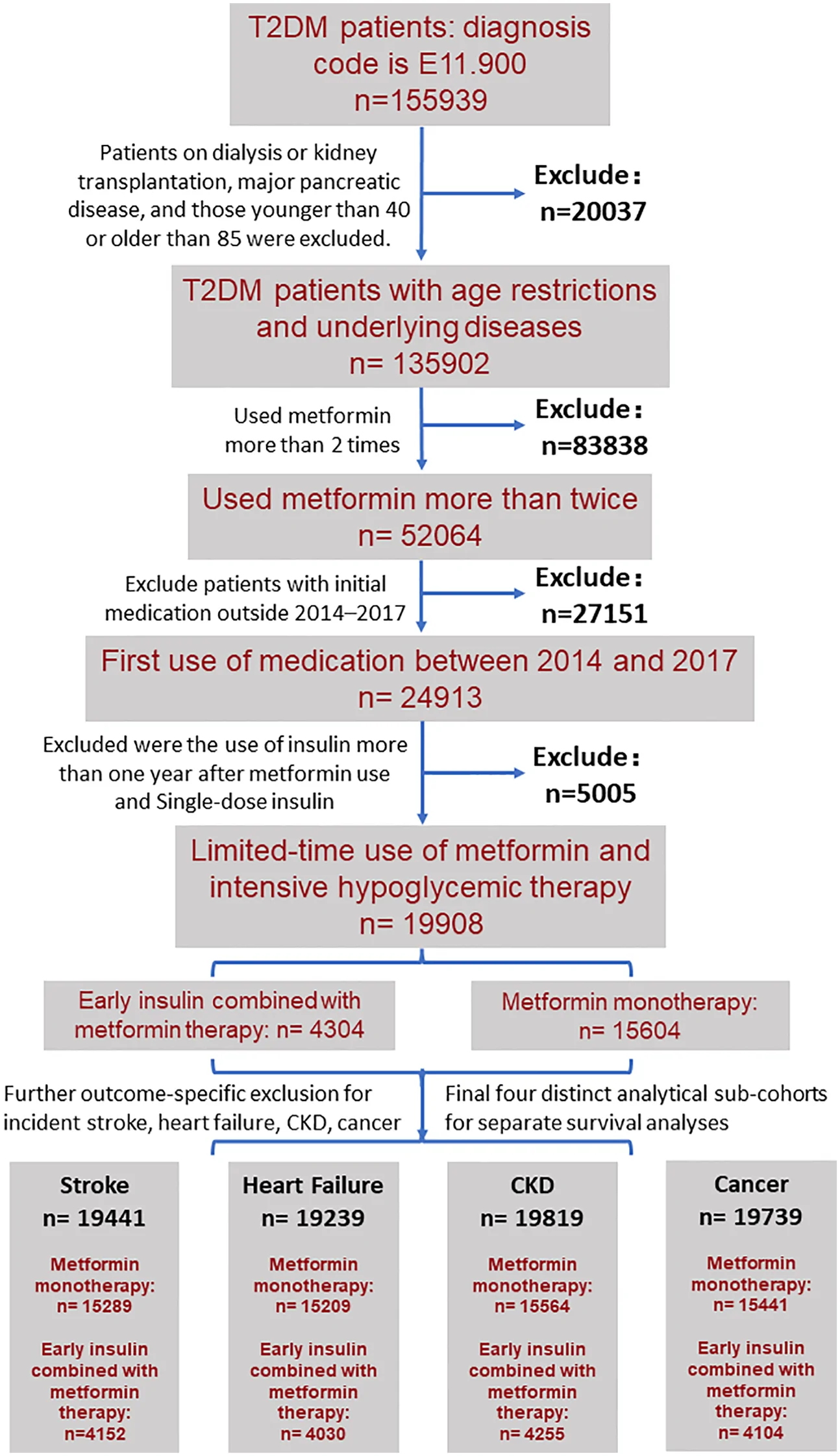
Flowchart for construction of the common parent cohort and the four outcome-specific analytic subcohorts among new users of metformin monotherapy and early insulin combined with metformin therapy in the Cheeloo LEAD database. The cohort-entry period was January 2014 through December 2017, with follow-up through December 2021. For each outcome-specific subcohort, patients with a baseline history of the corresponding outcome or with that outcome recorded within 30 days after t0 were excluded before analysis.

The age distribution of participants was concentrated between 50 and 75 years and approximated a normal distribution, with the peak age at diagnosis occurring between 60 and 70 years.

Before matching, analysis of outcome-specific incidence rates by sex revealed several notable patterns (**Supplementary Table S1**). For stroke events, females exhibited a higher incidence density than males. The average incidence density of female was 1.06 per 10,000 person-days, and cases were predominantly concentrated in the 80–85 age group. In contrast, males had an average incidence density of 0.94 per 10,000 person-days, with a similar age concentration (80–85 years).

Among patients who developed heart failure, females also had a higher incidence density. The incidence density of female was 0.85 per 10,000 person-days, and the majority of events occurred in the 75–79 age range. For males, the incidence density was slightly lower at 0.81 per 10,000 person-days, with peak incidence in the 80–85 age group.

For chronic kidney disease (CKD), males had a higher incidence density than females. The incidence density of male was 0.21 per 10,000 person-days, while female patients had an incidence density of 0.15 per 10,000 person-days. In both sexes, the highest frequency of CKD cases was observed in the 80–85 age group.

In the case of cancer outcomes, incidence density was again higher among males. Male patients had an incidence density of 0.44 per 10,000 person-days, with cases primarily occurring between the ages of 80 and 85. Female patients a lower incidence density of 0.37 per 10,000 person-days, with the peak incidence observed in the 80–85 age group.

After the common parent cohort was established, baseline covariates were extracted and screened using L1-regularized logistic regression. Outcome-specific baseline exclusions were then applied, and patients with the corresponding outcome recorded during the 30 days after t0 were excluded from that analysis. The final analytic sample sizes were 19,441 for stroke, 19,239 for heart failure, 19,819 for CKD, and 19,545 for cancer.

Baseline characteristics of the study population stratified by clinical outcomes are detailed in **Supplementary Table S2**. Before matching, significant differences were observed in age and gender distributions across the outcome groups (P<0.05).

### 3.2 Evaluation of the propensity score matching model

#### 3.2.1 Preference score distribution

The distribution of preference scores between the Early insulin combined with metformin therapy group and the metformin monotherapy group indicated substantial overlap, with 51.4% to 53.2% of individuals sharing common preference score ranges. This suggests that a sufficient number of patients were comparable across treatment groups, supporting the validity of the matching process. The area under the curve (AUC) for the propensity score model was 0.80, indicating good discriminative ability of baseline covariates in predicting treatment assignment (shown in **Supplementary Figure S1**).

#### 3.2.2 Standardized mean difference plot

The standardized mean difference (SMD) plot illustrates the balance of high-dimensional covariates before and after propensity score matching. Each point represents the SMD of a covariate, reflecting the degree of imbalance between each group. Before matching, considerable imbalance was observed, with SMD values ranging from approximately 0 to 0.3.

After matching, all covariates had SMD values below 0.1, indicating that propensity score matching effectively improved covariate balance. The substantial reduction in standardized differences suggests that the two groups achieved improved comparability, supporting the robustness of the matching process (shown in **Supplementary Figure S2**).

#### 3.2.3 Description of baseline covariates after matching

After propensity score matching, measured covariates showed improved balance between treatment groups in each outcome-specific analytic subcohort. **Table 1** presents selected baseline characteristics and balance diagnostics for the stroke, heart failure, CKD, and cancer subcohorts. Patients with a baseline history of the panel-specific outcome had been excluded before the corresponding analysis; therefore, the corresponding outcome is not listed as a baseline covariate in its own panel.

**Table 1:** Baseline characteristics and covariate balance before and after propensity score matching in the four outcome-specific analytic subcohorts.

Outcome	Baseline variables	Before matching				After matching			
		Metformin monotherapya (%)	Early insulin combined with metforminb (%)	*p*	SMD	Metformin monotherapya (%)	Early insulin combined with metforminb (%)	*p*	SMD
Stroke	gender	50	47.4	0.003	48.4	47.1	0.238	0.027	0.027
	Hypertension	59.1	53.2	<0.001	0.12	53.6	52.8	0.495	0.016
	Acute myocardial infarction	2.5	4.1	<0.001	0.09	3.7	3.8	0.905	0.004
	Cor pulmonale	0.8	1.7	<0.001	0.079	1.3	1.3	0.921	0.005
	Cardiomyopathy	0.8	1.7	<0.001	0.079	1.3	1.3	0.921	0.005
	Acute upper respiratory tract infection	6.4	6.9	0.244	0.021	6.8	6.8	1	<0.001
	Asthma	1.6	1.7	0.781	0.006	1.4	1.5	0.637	0.013
	Digestive system diseases	32.4	37.9	<0.001	0.115	35.3	36	0.538	0.014
	Cirrhosis	0.5	1.4	<0.001	0.09	0.9	1.1	0.426	0.021
	Pancreatic diseases	0.4	1.2	<0.001	0.09	0.7	0.8	0.79	0.009
	Other diseases of the digestive system	1.1	2.7	<0.001	0.117	1.6	1.7	0.929	0.004
	Hepatitis	1.1	2.2	<0.001	0.091	1.5	1.8	0.422	0.02
	Disorders of bone density and structure	2.4	3.7	<0.001	0.078	3.1	3	0.948	0.003
	Pregnancy, childbirth and postpartum period	0.7	1.5	<0.001	0.077	1.2	1.2	1	<0.001
	Fungal diseases	0.6	0.7	0.57	0.012	0.5	0.6	0.65	0.014
	Nephritis and kidney disease	2.8	5.1	<0.001	0.121	3.8	4.4	0.21	0.03
	Chronic kidney disease	0.8	1.7	<0.001	0.083	1.2	1.4	0.548	0.016
	Obesity and other overnutrition	0.4	1.1	<0.001	0.079	0.7	0.8	0.693	0.012
	Metabolic disorders	16.4	20.2	<0.001	0.097	18.1	19	0.307	0.024
Heart Failure	gender	50	47.2	0.001	0.057	48.8	46.8	0.08	0.041
	Hypertension	59	52.5	<0.001	0.131	51.8	52.2	0.73	0.008
	Acute myocardial infarction	2.3	3.3	<0.001	0.06	3.1	3.1	0.95	0.003
	Cor pulmonale	0.7	1.3	<0.001	0.063	1	1.1	0.82	0.008
	Ischemic stroke	20.7	18.6	0.003	0.054	18.3	18.7	0.7	0.01
	Acute upper respiratory tract infection	6.3	6.6	0.57	0.011	6.2	6.5	0.67	0.011
	Digestive system diseases	32.1	37.6	<0.001	0.116	35	35.6	0.63	0.012
	Pancreatic diseases	0.4	1.1	<0.001	0.087	0.6	0.7	0.77	0.01
	hepatitis	1.1	2.3	<0.001	0.095	1.5	1.6	0.85	0.006
	Nephritis and kidney disease	2.7	4.9	<0.001	0.117	3.9	4.3	0.49	0.017
	Kidney disease	2.1	4	<0.001	0.115	3.2	3.4	0.70	0.01
	Chronic kidney disease	0.7	1.6	<0.001	0.078	1.1	1.2	0.75	0.01
	Urogenital system diseases	10.1	12.6	<0.001	0.078	11	11.8	0.29	0.025
	Other malignant tumors	2.6	5.6	<0.001	0.153	3.6	4.2	0.29	0.026
	Malignant tumors of endocrine glands	0.4	0.5	0.74	0.008	0.4	0.5	0.74	0.012
	Other anemia	1.8	3.9	<0.001	0.124	2.8	2.9	0.73	0.01
	Uterine malignancy	0.3	0.7	<0.001	0.065	0.4	0.5	0.74	0.012
	Urinary system diseases	4.3	5.8	<0.001	0.068	5.1	5.5	0.45	0.018
	Obesity and other overnutrition	0.4	1.1	<0.001	0.08	0.7	0.8	0.69	0.012
	Metabolic disorders	16.5	20.2	<0.001	0.095	18.8	19.1	0.80	0.007
Chronic Kidney Disease	gender	50	47.1	0.001	0.057	47	46.8	0.88	0.004
	Cardiovascular disease	75.6	70	<0.001	0.124	70.6	69.8	0.45	0.018
	Hypertension	59.3	53.1	<0.001	0.124	53.5	52.9	0.64	0.011
	Acute myocardial infarction	2.4	4	<0.001	0.087	3.6	3.8	0.77	0.008
	Cor pulmonale	0.8	1.6	<0.001	0.073	1.2	1.3	0.68	0.011
	Cardiomyopathy	0.2	0.4	0.011	0.042	0.3	0.4	0.7	0.013
	Ischemic stroke	20.7	18.8	0.007	0.047	17.9	18.9	0.29	0.025
	Digestive system diseases	32.4	37.8	<0.001	0.114	35.8	36	0.87	0.004
	Cirrhosis	0.5	1.4	<0.001	0.086	0.9	1.1	0.43	0.02
	Other diseases of the digestive system	1.1	2.6	<0.001	0.113	1.7	1.7	0.93	0.004
	hepatitis	1	2.1	<0.001	0.086	1.4	1.8	0.25	0.028
	Urinary system diseases	4.3	5.9	<0.001	0.072	5.2	5.3	0.84	0.006
	Digestive system cancer	1.3	3.9	<0.001	0.162	2.2	2.2	0.88	0.005
	Malignant tumors	5.1	10.2	<0.001	0.193	7.2	7.6	0.49	0.016
	Urinary tract malignancies	0.4	0.4	0.74	0.008	0.5	0.4	0.86	0.008
	Malignant tumors of endocrine glands	0.4	0.5	0.72	0.008	0.4	0.5	0.60	0.016
	Uterine malignancy	0.3	0.7	<0.001	0.057	0.5	0.5	0.87	0.007
	anemia	2.4	4.9	<0.001	0.137	3.2	3.7	0.30	0.025
	Obesity and other overnutrition	0.4	1.1	<0.001	0.08	0.7	0.7	1	<0.001
	Metabolic disorders	16.5	20	<0.001	0.09	17.8	18.8	0.25	0.027
Cancer	gender	50	46.8	<0.001	0.065	47.3	46.7	0.65	0.011
	Hypertension	59.3	53.1	<0.001	0.124	53.1	52.7	0.803	0.006
	Acute myocardial infarction	2.5	4.2	<0.001	0.096	3.9	3.8	0.953	0.003
	Cor pulmonale	0.8	1.7	<0.001	0.08	1.1	1.4	0.355	0.023
	Cardiomyopathy	0.2	0.5	0.002	0.049	0.3	0.4	0.71	0.013
	Ischemic stroke	20.8	18.9	0.009	0.047	18	18.9	0.29	0.025
	Acute upper respiratory tract infection	6.4	6.7	0.44	0.014	6.2	6.6	0.43	0.019
	asthma	1.6	1.5	0.892	0.004	1.2	1.4	0.427	0.02
	Peptic ulcer	1.4	1.3	0.569	0.011	1.2	1.3	0.758	0.009
	Gastric/duodenal ulcer	1.6	1.5	0.915	0.003	1.4	1.5	0.85	0.006
	Pancreatic diseases	0.4	1.1	<0.001	0.082	0.7	0.7	1	<0.001
	Other diseases of the digestive system	1.1	2.5	<0.001	0.108	1.7	1.8	0.931	0.004
	hepatitis	1	2	<0.001	0.08	1.3	1.6	0.216	0.03
	Disorders of bone density and structure	2.3	3.7	<0.001	0.077	2.8	3	0.686	0.011
	Nephritis	1.8	3.5	<0.001	0.107	2.6	3	0.337	0.023
	Fungal diseases	0.6	0.6	0.819	0.006	0.5	0.6	0.634	0.014
	Nephritis and kidney disease	2.8	5	<0.001	0.116	4	4.3	0.53	0.016
	Urinary system diseases	4.3	5.8	<0.001	0.068	5.1	5.5	0.448	0.018
	Obesity and other overnutrition	0.4	1.1	<0.001	0.08	0.7	0.8	0.693	0.012
	Metabolic disorders	16.5	20.2	<0.001	0.095	18.8	19.1	0.794	0.007

Each panel represents a separate outcome-specific analytic subcohort. Patients with a baseline history of the corresponding panel outcome were excluded before propensity score matching and Cox analysis. Conditions shown in other panels were retained as potential confounders for those outcomes.

a, Metformin monotherapy; b, Early insulin combined with metformin therapy.

#### 3.2.4 Kaplan–Meier survival curves

Kaplan–Meier survival curves were plotted after propensity score matching (**Figure 3**). The curves provide descriptive comparisons of event-free survival between the two treatment groups within each outcome-specific matched subcohort. The corresponding log-rank P values were below 0.05 for the four outcomes.

**Figure 3:**
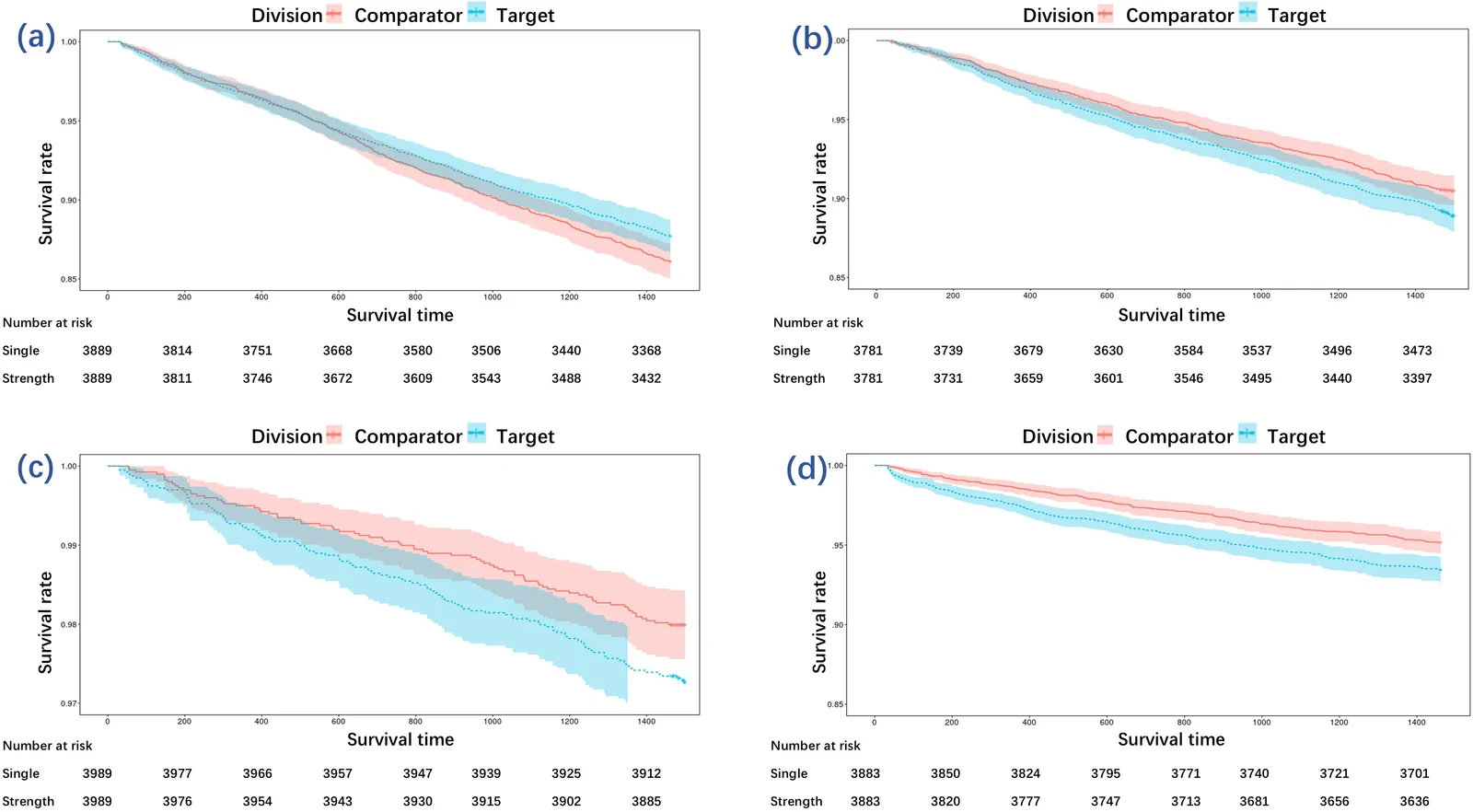
Kaplan–Meier curves after propensity score matching for **(a)** stroke, **(b)** heart failure, **(c)** chronic kidney disease, and **(d)** cancer among new users in the Cheeloo LEAD database. In the legacy plotting labels, “Single” and “Comparator” denote Metformin monotherapy, whereas “Strength,” “Target,” and “Intensive glucose control” denote Early insulin combined with metformin therapy.

Event-free survival was lower in the early insulin combined with metformin therapy group than in the metformin monotherapy group for heart failure, CKD, and cancer, whereas the stroke curve showed the opposite pattern. These descriptive curves were not interpreted as evidence that matching eliminated all residual confounding.

#### 3.2.5 Long-term association estimates for early insulin combined with metformin therapy

In the L1-regularized propensity score-matched subcohorts, Cox proportional hazards models were used to estimate longitudinal associations (**Figure 4**). Compared with metformin monotherapy, early insulin combined with metformin therapy was associated with a lower hazard of stroke (HR 0.88, 95% CI 0.78–0.99) and higher hazards of heart failure (HR 1.18, 95% CI 1.02–1.36), CKD (HR 1.37, 95% CI 1.03–1.83), and cancer (HR 1.37, 95% CI 1.14–1.66). All models satisfied the proportional hazards assumption.

**Figure 4:**
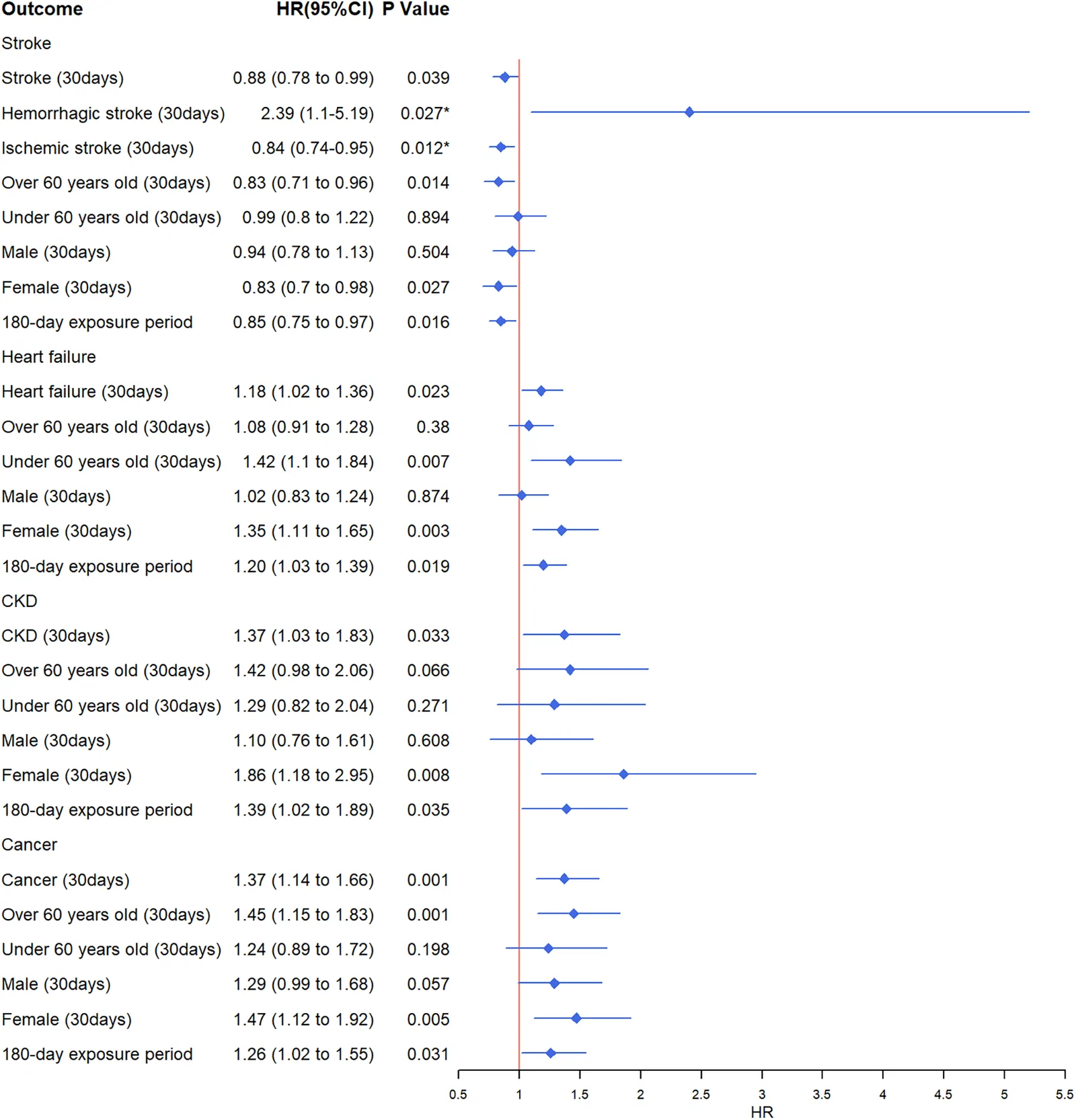
Forest plot of Cox proportional hazards model estimates from exploratory age- and sex-stratified analyses and the 180-day outcome-exclusion sensitivity analysis among new users of metformin monotherapy and early insulin combined with metformin therapy. In the 180-day sensitivity analysis, the post-t0 outcome-exclusion window was extended from 30 to 180 days while retaining the same t0, treatment-strategy definition, and follow-up start. Formal interaction tests were not performed; subgroup-specific estimates should not be interpreted as formal comparisons between subgroups.

In exploratory stroke subtype analyses, compared with metformin monotherapy, early insulin combined with metformin therapy was associated with a lower hazard of ischemic stroke (HR 0.84, 95% CI 0.74–0.95) and a higher hazard of hemorrhagic stroke (HR 2.39, 95% CI 1.10–5.19). Because hemorrhagic stroke events were relatively few and the confidence interval was wide, this estimate should be interpreted as exploratory.

### 3.3 Subgroup analyses

In exploratory age-stratified analyses, the inverse association with stroke was observed among participants aged ≥60 years (HR 0.83, P = 0.014), whereas the estimate among those aged <60 years was close to the null (HR 0.99, P = 0.894). In contrast, the higher heart failure hazard was observed among participants aged <60 years (HR 1.42, P = 0.007), while the estimate among those aged ≥60 years was not statistically significant (HR 1.08, P = 0.38).

Sex-stratified estimates are presented descriptively in **Figure 4**. No formal interaction tests were performed for age or sex; therefore, numerical variation and differences in statistical significance between subgroup-specific estimates were not interpreted as evidence that the longitudinal associations differed across subgroups.

### 3.4 Sensitivity analyses

In the sensitivity analysis, the post-t0 outcome-exclusion window was extended from 30 to 180 days while follow-up continued to begin at the strategy-specific t0. The resulting association estimates were broadly consistent with the primary analyses.

## 4 Discussion

Using an active-comparator new-user cohort design, this study compared longitudinal outcomes between two clinically relevant early treatment strategies for newly diagnosed T2DM: metformin monotherapy and early insulin combined with metformin therapy.

Compared with metformin monotherapy, early insulin combined with metformin therapy was associated with a lower hazard of stroke and higher hazards of heart failure, CKD, and cancer. These findings describe longitudinal associations in routine care and should be interpreted in light of residual confounding by baseline disease severity and treatment indication.

Both observational studies and *post hoc* analyses of randomized clinical trials have examined the cardiovascular effects of metformin and insulin therapies. As a recently emerging strategy for the treatment of type 2 diabetes mellitus, early insulin-based therapy has been reported to maintain near-normal blood glucose levels and potentially restore impaired pancreatic β-cell function (28). It has also been shown to effectively control blood glucose, induce glycemic remission, and alleviate insulin resistance (29).

With regard to each of the intermediate and long-term complication outcomes:

Regarding stroke outcomes, a historical prospective cohort study based on the Clalit Health Services (CHS) electronic medical record database found a trend toward increased stroke risk among insulin users with non-valvular atrial fibrillation (NVAF) and coexisting diabetes mellitus (DM) (30). In contrast, the study by Bonnet, F., and Scheen et al. reported a neutral effect of insulin use at standard dosages on the risk of stroke (31). These findings are consistent with the results of the present study. In exploratory subtype analyses, early insulin combined with metformin therapy was associated with a lower hazard of ischemic stroke and a higher hazard of hemorrhagic stroke than metformin monotherapy; the hemorrhagic estimate was based on relatively few events and had a wide confidence interval. For ischemic stroke, which showed a trend toward a protective effect, a study by Lioutas et al. demonstrated that intranasal insulin administration improved survival and neurological function in a mouse model of ischemic stroke (32). This suggests that insulin may exert neuroprotective, anti-inflammatory, and anti-apoptotic effects on brain tissue (33). These findings are further supported by mechanistic evidence indicating that insulin and its analogs (e.g., IGF-1) may facilitate stroke recovery by enhancing cerebral energy metabolism, promoting neuroplasticity, inhibiting thrombosis, and inducing vasodilation (32). In contrast, hemorrhagic stroke, which follows a different pathophysiological mechanism, has been associated with poorer outcomes during intensive insulin therapy. Clinical observational studies have shown that such therapy may increase the risk of hypoglycemia, potentially leading to hemorrhagic transformation and worse prognoses (34). In addition, insulin resistance has been positively associated with hemorrhagic transformation in non-diabetic patients receiving intravenous thrombolysis for ischemic stroke (35).

With respect to heart failure outcomes, the ORIGIN trial demonstrated a neutral effect of insulin therapy on hospitalization for heart failure (36). However, that study focused on insulin monotherapy rather than early insulin combined with metformin therapy, and included participants with impaired glucose tolerance or impaired fasting glucose. Other studies have reported that, compared with non-insulin therapies, insulin treatment may increase the risks of mortality, hospitalization, and rehospitalization in patients with diabetes and heart failure (37). Furthermore, research by Herman et al. showed a dose-dependent association between insulin use and increased cardiovascular risk and mortality in patients with type 2 diabetes, suggesting a heightened susceptibility to heart failure (38). These findings may help explain the elevated mortality observed in the Early insulin combined with metformin therapy group in the present study.

For chronic kidney disease (CKD) outcomes, although there is currently no direct evidence indicating that early insulin combined with metformin therapy impairs renal function, existing studies have suggested that insulin resistance may contribute to increased susceptibility to CKD among patients with diabetes (39). This may be relevant given that early insulin treatment is often initiated in populations with poor glycemic control, where insulin resistance is more prevalent.

Regarding cancer outcomes, several studies have reported that insulin use is associated with an increased incidence of certain types of cancer (40, 41), a finding that aligns with the results of the present study. In addition, meta-analyses have indicated that insulin therapy may elevate the risk of site-specific malignancies, such as pancreatic and colorectal cancers (42), as well as hepatocellular carcinoma (43). One proposed mechanism is that insulin may promote tumor growth through activation of the insulin-like growth factor-1 (IGF-1) receptor (44). This pathophysiological pathway may partly explain the higher hazard ratio observed for cancer in the Early insulin combined with metformin therapy group.

Subgroup analyses were conducted by sex and age for each outcome. In the sex-based subgroup analysis, the subgroup-specific estimates showed numerical variation between women and men; however, no formal interaction tests were performed. Previous literature has described sex-specific physiological responses of organ systems to type 2 diabetes mellitus (45–48). Furthermore, previous studies have demonstrated that the adverse metabolic effects of diabetes tend to be more pronounced in women than in men (49).

In the exploratory age-stratified analyses, the inverse stroke association was observed among participants aged ≥60 years, whereas the higher heart failure hazard was observed among those aged <60 years. No formal interaction tests were performed. Previous studies have suggested that age-related differences in diabetes outcomes may reflect the combined influence of long-standing poor glycemic control, cumulative burden of chronic diseases, age-related physiological changes, and declining immune function (50).

The exploratory heart failure estimate was higher among participants aged <60 years than among those aged ≥60 years; however, without interaction testing, this numerical pattern does not establish an age-specific difference. Data from the Swedish National Diabetes Register and the Joint Asia Diabetes Evaluation (JADE) program suggest that individuals diagnosed with diabetes before the age of 40 have a higher risk burden and experience greater loss of life-years due to cardiovascular disease (51). These observations provide clinical context but do not establish that one treatment strategy is preferable for a specific age group.

In addition, a 180-day post-t0 outcome-exclusion window was applied as a sensitivity analysis to test the robustness of the findings. The results obtained under the extended outcome-exclusion window were consistent with those of the primary 30-day analysis, aligning with previous research (52) and further reinforcing the reliability of our study outcomes.

## 5 Strengths and weaknesses of this study

### 5.1 Strengths

This study focused on type 2 diabetes mellitus (T2DM), a common disease with complex complications, and utilized regional population-based data from China to provide additional real-world evidence supporting early and rational treatment strategies for diabetes. An active-comparator new-user design was employed to closely emulate the structure of randomized controlled trials (RCTs), while the use of a large-scale, regionally representative electronic health record database supported the relevance of the findings to routine care in the study region.

L1-regularized propensity score matching improved balance in a high-dimensional set of measured baseline characteristics. Age- and sex-stratified estimates were retained as exploratory analyses of consistency and were not used to infer subgroup heterogeneity or preferential treatment suitability.

### 5.2 Limitations

Although the large-scale electronic health record data used in this study were derived from multiple medical centers, the geographic coverage was limited to Shandong Province, and evidence from other regions of China is lacking. Moreover, as the study relied primarily on hospital-based EHR data, medication records for patients in primary care and community settings may have been incomplete or subject to misclassification due to unrecorded prescriptions or medications purchased by others on behalf of the patient (53). This may have led to the exclusion of some patients with T2DM who were not accurately identified as long-term medication users.

Although L1-regularized propensity score matching improved balance in measured baseline variables, residual confounding cannot be excluded. In addition, T2DM onset was defined by the first recorded diagnosis in the database, which may not coincide with the true date of disease onset.

No laboratory or anthropometric measure of baseline disease severity, including HbA1c, fasting blood glucose, BMI, eGFR, urinary protein, or urine albumin, was incorporated into the analytic dataset because these data were substantially incomplete and were recorded at non-uniform time points. Consequently, differences in baseline glycemic and metabolic severity between the treatment groups could not be fully quantified. Although the exposure definition was informed by the clinical indications described in the Chinese guideline, this design consideration could not replace individual-level quantitative adjustment (3). Residual confounding by baseline disease severity and treatment indication may therefore have influenced the observed longitudinal associations, particularly for heart failure, CKD, and cancer. These findings should be interpreted cautiously.

Mortality ascertainment in the analytic data relied on available hospital death records, and deaths occurring outside participating hospitals may not have been completely captured. Participants who died before the target outcome were right-censored at death. This approach standardizes person-time calculation but cannot remove informative censoring or baseline severity differences and may have contributed to overestimation of some observed associations.

The age- and sex-stratified analyses were exploratory, and no formal interaction tests were performed. Numerical variation between subgroups should therefore not be interpreted as evidence that the associations differed by age or sex. In addition, the findings apply to patients initiating early insulin combined with metformin therapy and should not be generalized to patients initiating insulin monotherapy or strategies without concurrent metformin.

While a specific chart-review validation study for the ICD-10 codes in this cohort was not performed, the reliability of the data is supported by the infrastructure of the Cheeloo LEAD database. This database utilizes the Observational Medical Outcomes Partnership (OMOP) Common Data Model (CDM), which employs rigorous Extract, Transform, and Load (ETL) processes to map source diagnoses to standardized vocabularies. This standardization ensures semantic consistency and data quality across the heterogeneous electronic health records.

## 6 Conclusion

In this real-world EHR cohort, early insulin combined with metformin therapy, compared with metformin monotherapy, showed a lower hazard of stroke and higher hazards of heart failure, CKD, and cancer. These longitudinal associations do not establish treatment effects and should be interpreted cautiously because laboratory measures of baseline severity were unavailable and residual confounding by treatment indication may remain. Further studies with more complete clinical severity measures are warranted.

## Data Availability

The data used in this study are not publicly available due to privacy and ethical restrictions involving routinely collected healthcare data. Access to the data may be granted upon reasonable request and approval by the corresponding institution and ethics committee. Requests to access these datasets should be directed to FX, xuefzh@sdu.edu.cn.
