# Supplementary material for: Early insulin combined with metformin therapy and long-term risks of cardiovascular disease, kidney disease, and cancer in newly diagnosed type 2 diabetes: a real-world cohort study in China
Source: Front Endocrinol (Lausanne). 2026 Sep 21;17:1877839. doi: 10.3389/fendo.2026.1877839 (PMC13635304; doi:10.3389/fendo.2026.1877839)
Supplement: Supplementary file 1 [file DataSheet1.pdf]

The RECORD statement – checklist of items, extended from the STROBE statement, that should be reported in observational studies using routinely collected health data.

	Item No.	STROBE items	Location in manuscript where items are reported	RECORD items	Location in manuscript where items are reported
**Title and abstract**					
	1	(a) Indicate the study's design with a commonly used term in the title or the abstract (b) Provide in the abstract an informative and balanced summary of what was done and what was found	Title: p1, lines 1–4; Abstract: p2, lines 24–45; Keywords: p2, lines 46–48	RECORD 1.1: The type of data used should be specified in the title or abstract. When possible, the name of the databases used should be included.<br><br>RECORD 1.2: If applicable, the geographic region and timeframe within which the study took place should be reported in the title or abstract.<br><br>RECORD 1.3: If linkage between databases was conducted for the study, this should be clearly stated in the title or abstract.	Abstract—Methods: p2, lines 28–37; database name "Cheeloo LEAD": p2, line 28.<br><br>Abstract—Methods: p2, lines 28–31; setting/timeframe "Shandong, China; 2014–2021": p2, lines 28–30.<br><br>Data linkage not stated in title/abstract; linkage described in Methods—Data sources: p4, lines 88–91.
**Introduction**					
Background rationale	2	Explain the scientific background and rationale for the investigation being reported	Introduction: p3, lines 51–73		

Objectives	3	State specific objectives, including any prespecified hypotheses	Introduction: p3, lines 74–80		
**Methods**					
Study Design	4	Present key elements of study design early in the paper	Methods—Data sources: p4, lines 82–99; Cohort design: p4–p5, lines 104–148		
Setting	5	Describe the setting, locations, and relevant dates, including periods of recruitment, exposure, follow-up, and data collection	Setting and dates: p4, lines 84–99; cohort entry/follow-up: p5, lines 120–148		
Participants	6	*(a) Cohort study* - Give the eligibility criteria, and the sources and methods of selection of participants. Describe methods of follow-up<br>*Case-control study* - Give the eligibility criteria, and the sources and methods of case ascertainment and control selection. Give the rationale for the choice of cases and controls<br>*Cross-sectional study* - Give the eligibility criteria, and the sources and methods of selection of participants<br><br>*(b) Cohort study* - For matched studies, give matching criteria and number of exposed and unexposed<br>*Case-control study* - For matched studies, give matching criteria and the number of controls per case	Eligibility and selection: p5, lines 127–143; follow-up definition: p5, lines 142–143; matching: p6, lines 174–190; participant flow: p7, lines 220–227	RECORD 6.1: The methods of study population selection (such as codes or algorithms used to identify subjects) should be listed in detail. If this is not possible, an explanation should be provided.<br><br>RECORD 6.2: Any validation studies of the codes or algorithms used to select the population should be referenced. If validation was conducted for this study and not published elsewhere, detailed methods and results should be provided.<br><br>RECORD 6.3: If the study involved linkage of databases, consider use of a flow diagram or other graphical display to demonstrate the data linkage process, including the number of individuals with linked data at each stage.	Population selection codes/algorithms: T2DM ICD-10 E11.900 p5, lines 127–130; exclusion criteria p5, lines 135–141.<br><br>No separate validation study reported; ICD-code validation limitation discussed: p15, lines 442–447.<br><br>Database linkage described in Methods: p4, lines 88–91; study flow diagram for cohort selection: p7, lines 220–227.

Variables	7	Clearly define all outcomes, exposures, predictors, potential confounders, and effect modifiers. Give diagnostic criteria, if applicable.	Exposure: p5, lines 149–159; outcomes: p5–p6, lines 160–164; covariates: p6, lines 165–173	RECORD 7.1: A complete list of codes and algorithms used to classify exposures, outcomes, confounders, and effect modifiers should be provided. If these cannot be reported, an explanation should be provided.	Outcome ICD-10 codes listed: p5–p6, lines 160–164; covariate code examples: p6, lines 165–173.
Data sources/ measurement	8	For each variable of interest, give sources of data and details of methods of assessment (measurement).<br>Describe comparability of assessment methods if there is more than one group	Data sources: p4, lines 84–99; outcome/covariate measurement: p5–p6, lines 160–173		
Bias	9	Describe any efforts to address potential sources of bias	Bias/design features: p4–p5, lines 104–148; confounder control and matching: p6, lines 174–190		
Study size	10	Explain how the study size was arrived at	Results—Cohort: p7, lines 220–224; outcome-specific cohorts: p8, lines 249–254		
Quantitative variables	11	Explain how quantitative variables were handled in the analyses. If applicable, describe which groupings were chosen, and why	Statistical analysis: p6–p7, lines 192–207		
Statistical methods	12	(a) Describe all statistical methods, including those used to control for confounding<br>(b) Describe any methods used to examine subgroups and interactions<br>(c) Explain how missing data were addressed	Statistical analysis: p6–p7, lines 192–209; subgroup/sensitivity analyses: p7, lines 204–207		

		(d) *Cohort study* - If applicable, explain how loss to follow-up was addressed<br>*Case-control study* - If applicable, explain how matching of cases and controls was addressed<br>*Cross-sectional study* - If applicable, describe analytical methods taking account of sampling strategy<br>(e) Describe any sensitivity analyses			
Data access and cleaning methods				RECORD 12.1: Authors should describe the extent to which the investigators had access to the database population used to create the study population.<br><br>RECORD 12.2: Authors should provide information on the data cleaning methods used in the study.	Data access: Author Contributors, p16, lines 469–472.<br><br>Data cleaning/standardization: p4, lines 96–99; complete records required: p5, lines 132–134; invalid identifiers excluded: p5, lines 139–140.
Linkage		..		RECORD 12.3: State whether the study included person-level, institutional-level, or other data linkage across two or more databases. The methods of linkage and methods of linkage quality evaluation should be provided.	Person-level deterministic linkage using encrypted unique ID: p4, lines 88–91.
**Results**					
Participants	13	(a) Report the numbers of individuals at each stage of the	Results—Cohort and Figure 2: p7, lines	RECORD 13.1: Describe in detail the selection of the persons included in the	Participant selection and

		study (*e.g.*, numbers potentially eligible, examined for eligibility, confirmed eligible, included in the study, completing follow-up, and analysed)<br>(b) Give reasons for non-participation at each stage.<br>(c) Consider use of a flow diagram	220–227; outcome-specific cohorts: p8, lines 249–254	study (*i.e.,* study population selection) including filtering based on data quality, data availability and linkage. The selection of included persons can be described in the text and/or by means of the study flow diagram.	filtering: p5, lines 127–143; Results—Cohort and Figure 2: p7, lines 220–227.
Descriptive data	14	(a) Give characteristics of study participants (*e.g.*, demographic, clinical, social) and information on exposures and potential confounders<br>(b) Indicate the number of participants with missing data for each variable of interest<br>(c) *Cohort study* - summarise follow-up time (*e.g.*, average and total amount)	Baseline/descriptive data: p7–p8, lines 220–257; Table 1: p9–p11, lines 284–286		
Outcome data	15	*Cohort study* - Report numbers of outcome events or summary measures over time<br>*Case-control study* - Report numbers in each exposure category, or summary measures of exposure<br>*Cross-sectional study* - Report numbers of outcome events or summary measures	Outcome data/KM curves/main outcomes: p11, lines 287–309		
Main results	16	(a) Give unadjusted estimates and, if applicable, confounder-adjusted estimates and their precision (e.g., 95% confidence interval). Make clear which confounders were adjusted for and why they were included	Main results: p11, lines 301–309; Figure 4: p12, lines 325–328		

		(b) Report category boundaries when continuous variables were categorized<br>(c) If relevant, consider translating estimates of relative risk into absolute risk for a meaningful time period			
Other analyses	17	Report other analyses done—e.g., analyses of subgroups and interactions, and sensitivity analyses	Subgroup analyses: p11–p12, lines 310–319; Sensitivity analyses: p12, lines 320–324		
**Discussion**					
Key results	18	Summarise key results with reference to study objectives	Discussion—key results: p12, lines 330–341		
Limitations	19	Discuss limitations of the study, taking into account sources of potential bias or imprecision. Discuss both direction and magnitude of any potential bias	Limitations: p14–p15, lines 428–447	RECORD 19.1: Discuss the implications of using data that were not created or collected to answer the specific research question(s). Include discussion of misclassification bias, unmeasured confounding, missing data, and changing eligibility over time, as they pertain to the study being reported.	Limitations of routinely collected data: p14–p15, lines 428–447.
Interpretation	20	Give a cautious overall interpretation of results considering objectives, limitations, multiplicity of analyses, results from similar studies, and other relevant evidence	Discussion interpretation: p12–p14, lines 342–413		
Generalisability	21	Discuss the generalisability (external validity) of the study results	Generalisability/strengths: p14, lines 415–427; limitations on geographic scope: p14–p15, lines 428–431		

Other Information					
Funding	22	Give the source of funding and the role of the funders for the present study and, if applicable, for the original study on which the present article is based	Funding: p16, lines 480–485		
Accessibility of protocol, raw data, and programming code		..		RECORD 22.1: Authors should provide information on how to access any supplemental information such as the study protocol, raw data, or programming code.	Data availability: p15–p16, lines 458–462; Supplementary material list: p16, lines 491–501.

*Reference: Benchimol EI, Smeeth L, Guttmann A, Harron K, Moher D, Petersen I, Sørensen HT, von Elm E, Langan SM, the RECORD Working Committee. The REporting of studies Conducted using Observational Routinely-collected health Data (RECORD) Statement. *PLoS Medicine* 2015; in press.

*Checklist is protected under Creative Commons Attribution (CC BY) license.
