# Supplementary material for: Early insulin combined with metformin therapy and long-term risks of cardiovascular disease, kidney disease, and cancer in newly diagnosed type 2 diabetes: a real-world cohort study in China
Source: Front Endocrinol (Lausanne). 2026 Sep 21;17:1877839. doi: 10.3389/fendo.2026.1877839 (PMC13635304; doi:10.3389/fendo.2026.1877839)
Supplement: Supplementary file 2 [file DataSheet2.pdf]

Fig s1: Propensity Score Preference Distributions of new users of metformin monotherapy and early insulin combined with metformin therapy in Cheeloo LEAD Database, included from January, 2014 to December, 2017 with follow-up extending to December 2021. (a) Stroke; (b) Heart Failure; (c) Chronic Kidney Disease (CKD); (d) Cancer

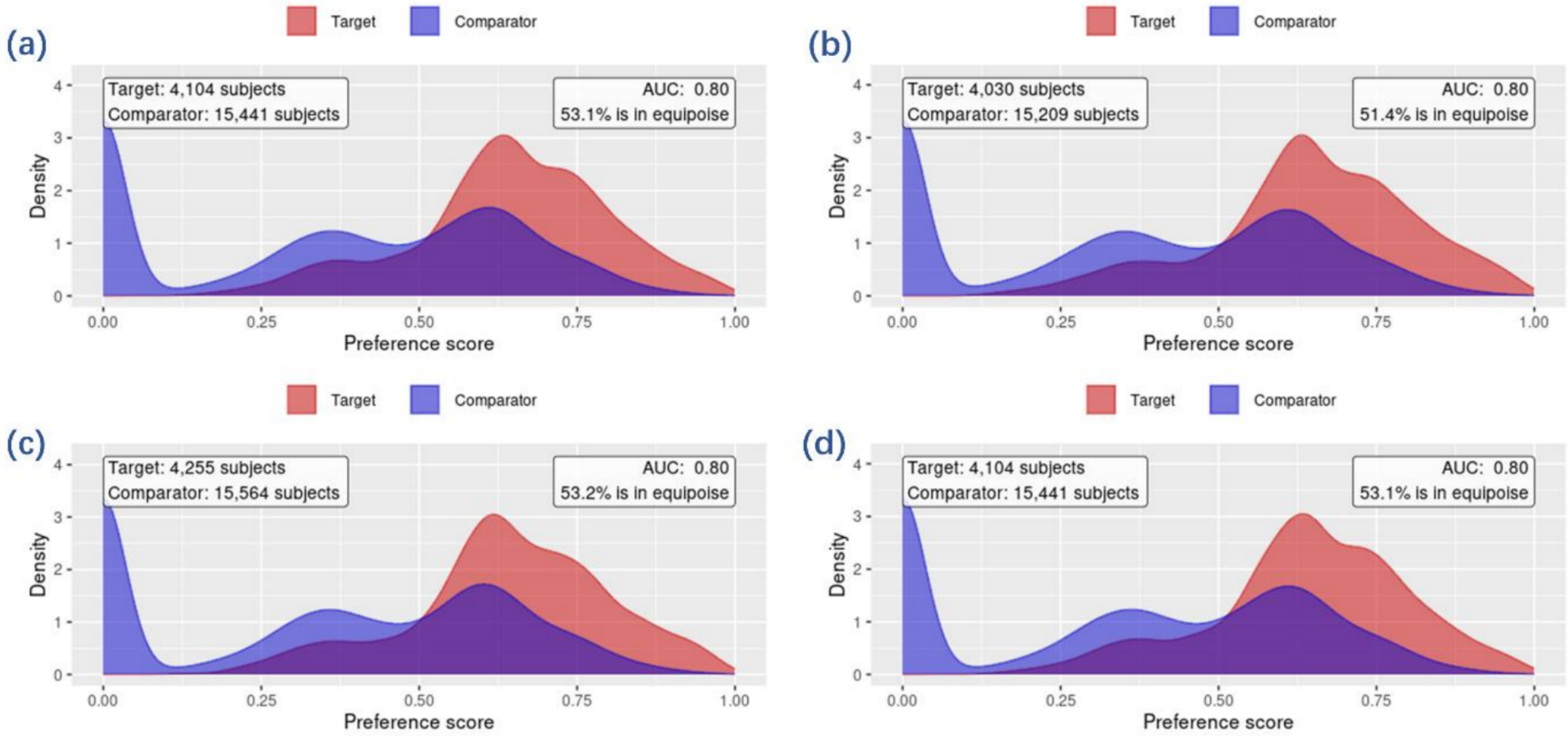

Fig s2. Standardized Mean Differences of Covariates Before and After Propensity Score Matching of new users of metformin monotherapy and early insulin combined with metformin therapy in Cheeloo LEAD Database, included from January, 2014 to December, 2017 with follow-up extending to December 2021. (a) Stroke; (b) Heart Failure; (c) Chronic Kidney Disease (CKD); (d) Cancer

(d) Cancer

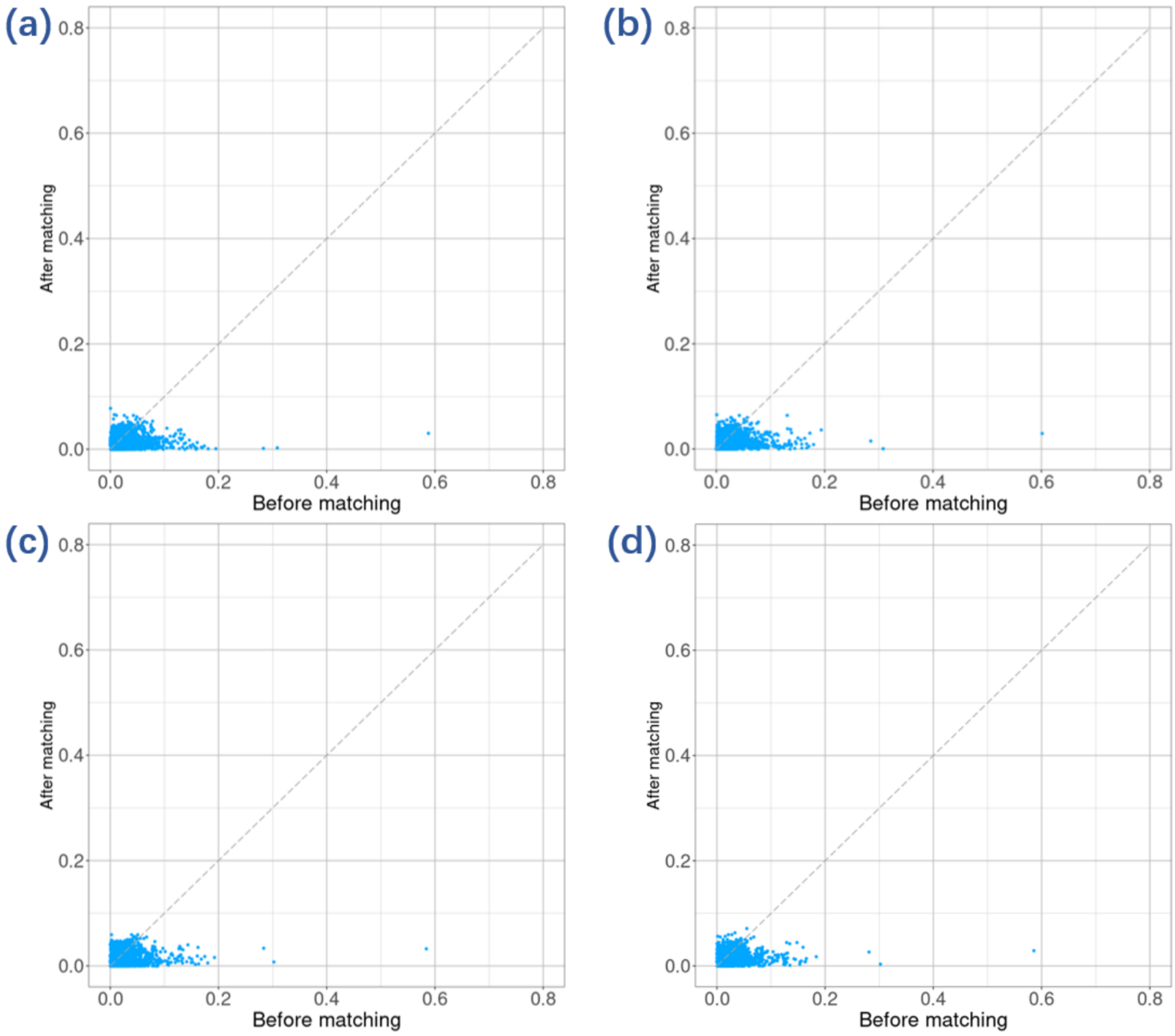

Table s1: Outcome density description table of new users of metformin monotherapy and early insulin combined with metformin therapy in Cheeloo LEAD Database, included from January, 2014 to December, 2017 with follow-up extending to December 2021. Table S1: Describe table of the incidence density of each outcome by age group.

incidence density of each outcome by age group.

stroke									
male					female				
age_duan	count	case	median_sf	incidence_density	age_duan	count	case	median_sf	incidence_density
40-44	461	25	1500	0.369808	40-44	288	15	1500	0.35396
45-49	852	55	1500	0.443467	45-49	502	35	1500	0.480462
50-54	1388	134	1500	0.680701	50-54	1072	108	1500	0.702445
55-59	1444	177	1500	0.870131	55-59	1301	154	1500	0.839382
60-64	1824	254	1500	0.999421	60-64	2000	284	1500	1.022073
65-69	1517	231	1500	1.095824	65-69	1866	325	1500	1.276541
70-74	1057	179	1500	1.239563	70-74	1408	260	1500	1.361716
75-79	727	142	1500	1.443351	75-79	957	173	1500	1.328877

80-85	343	67	1500	1.454129	80-85	434	88	1500	1.531711
total	9613	1264	1500	0.939281	total	9828	1442	1500	1.057592
heart failure									
male					female				
age_duan	count	case	median_sf	incidence_density	age_duan	count	case	median_sf	incidence_density
40-44	449	20	1500	0.30324	40-44	284	11	1500	0.262805
45-49	849	42	1500	0.337758	45-49	498	27	1500	0.37175
50-54	1392	116	1500	0.58064	50-54	1072	84	1500	0.543214
55-59	1440	138	1500	0.669981	55-59	1294	122	1500	0.659998
60-64	1810	203	1500	0.792803	60-64	1986	200	1500	0.706107
65-69	1502	198	1500	0.938418	65-69	1847	245	1500	0.947817
70-74	1039	171	1500	1.20447	70-74	1386	211	1500	1.097929
75-79	704	128	1500	1.339857	75-79	938	190	1500	1.505678
80-85	326	77	1500	1.809827	80-85	423	76	1500	1.345778
total	9511	1093	1500	0.813755	total	9728	1166	1500	0.850757
CKD									
male					female				
age_duan	count	case	median_sf	incidence_density	age_duan	count	case	median_sf	incidence_density
40-44	458	8	1500	0.117182	40-44	290	9	1500	0.210565
45-49	863	20	1500	0.156249	45-49	507	10	1500	0.133006
50-54	1411	37	1500	0.176727	50-54	1092	20	1500	0.123285
55-59	1472	34	1500	0.155728	55-59	1329	21	1500	0.10621
60-64	1867	64	1500	0.232288	60-64	2032	35	1500	0.115717
65-69	1550	43	1500	0.187601	65-69	1915	44	1500	0.154667
70-74	1080	30	1500	0.188168	70-74	1442	38	1500	0.17804
75-79	734	40	1500	0.371824	75-79	980	28	1500	0.193114
80-85	349	28	1500	0.560232	80-85	448	17	1500	0.2578
total	9784	304	1500	0.210281	total	10035	222	1500	0.149074
cancer									
male					female				
age_duan	count	case	median_sf	incidence_density	age_duan	count	case	median_sf	incidence_density
40-44	459	11	1500	0.16168	40-44	287	5	1500	0.117345
45-49	858	15	1500	0.117699	45-49	504	15	1500	0.201616
50-54	1397	62	1500	0.302438	50-54	1077	37	1500	0.233517
55-59	1453	67	1500	0.315213	55-59	1311	66	1500	0.346708
60-64	1832	134	1500	0.507809	60-64	1996	97	1500	0.33291
65-69	1521	122	1500	0.557399	65-69	1890	113	1500	0.410835
70-74	1059	94	1500	0.618511	70-74	1427	101	1500	0.49151
75-79	725	71	1500	0.686128	75-79	969	67	1500	0.479369
80-85	338	44	1500	0.934185	80-85	442	33	1500	0.515686
total	9642	620	1500	0.443382	total	9903	534	1500	0.370451

Table s3: Baseline characteristics of new users of metformin monotherapy and early insulin combined with metformin therapy in Cheeloo LEAD Database, included from January, 2014 to December, 2017 with follow-up extending to December 2021. Data are number (%) of users unless stated otherwise.

	Users	n	gender (n%) 0	gender (n%) 1	age (mean (SD))	y (n%) 0	y (n%) 1
Stroke	no	15289	7644 (50.0)	7645 (50.0)	62.64 (9.84)	13155 (86.0)	2134 (14.0)
	yes	4152	2184 (52.6)	1968 (47.4)	61.75 (10.27)	3648 (87.9)	504 (12.1)
	p		0.003		<0.001	0.003	
Heart failure	no	15209	7600 (50.0)	7609 (50.0)	62.61 (9.81)	13400 (88.1)	1809 (11.9)
	yes	4030	2128 (52.8)	1902 (47.2)	61.52 (10.20)	3580 (88.8)	450 (11.2)
	p		0.001		<0.001	0.21	
CKD	no	15564	7786 (50.0)	7778 (50.0)	62.67 (9.82)	15155 (97.4)	409 (2.6)
	yes	4255	2249 (52.9)	2006 (47.1)	61.82 (10.26)	4138 (97.3)	117 (2.7)
	p		0.001		<0.001	0.70	
Cancer	no	15441	7719 (50.0)	7722 (50.0)	62.66 (9.83)	14586 (94.5)	855 (5.5)
	yes	4104	2184 (53.2)	1920 (46.8)	61.71 (10.31)	3831 (93.3)	273 (6.7)
	p		<0.001		0.09	0.05	

Table S3: Baseline Characteristics and covariate balance test before and after propensity score matching between early insulin combined with metformin therapy and metformin monotherapy groups.

Stroke:

outcome	Baseline variables	ICD-10	level	before matching: Single drug[a]	before matching: Strengthen	before matching: p	before matching: SMD	after matching: Single drug[a]	after matching: Strengthen	after matching: p	after matching: SMD

stroke	Paroxysmal and episodic disorders	G40 – G47	0	13657 (89.3)	3764 (90.7)	0.014	0.044	3512 (90.3)	3524 (90.6)	0.671	0.011
			1	1632 (10.7)	388 (9.3)			377 (9.7)	365 (9.4)		
	Nerve: Nerve root and plexus disorders	G50 – G59	0	15044 (98.4)	4104 (98.8)	0.043	0.038	3837 (98.7)	3844 (98.8)	0.54	0.016
			1	245 (1.6)	48 (1.2)			52 (1.3)	45 (1.2)		
	Conjunctival diseases	H10 – H13	0	15027 (98.3)	4064 (97.9)	0.093	0.03	3826 (98.4)	3817 (98.1)	0.487	0.018
			1	262 (1.7)	88 (2.1)			63 (1.6)	72 (1.9)		
	Lens diseases	H25 – H28	0	14251 (93.2)	3828 (92.2)	0.025	0.039	3615 (93.0)	3597 (92.5)	0.458	0.018
			1	1038 (6.8)	324 (7.8)			274 (7.0)	292 (7.5)		
	septicemia	A40 – A41	0	15270 (99.9)	4138 (99.7)	0.006	0.044	3882 (99.8)	3881 (99.8)	1	0.006
			1	19 (0.1)	14 (0.3)			7 (0.2)	8 (0.2)		
	Other diseases of the eye and its adnexa	H55 – H59	0	15147 (99.1)	4109 (99.0)	0.59	0.011	3852 (99.0)	3851 (99.0)	1	0.003
			1	142 (0.9)	43 (1.0)			37 (1.0)	38 (1.0)		
	Sensory disorders (ear)	H60 – H95	0	14867 (97.2)	4035 (97.2)	0.883	0.004	3797 (97.6)	3783 (97.3)	0.349	0.023
			1	422	117			92	106		

				(2.8)	(2.8)			(2.4)	(2.7)		
	Cardiovascular disease	I00–I15, I20–I28, I30–I51, I60–I79	0	3794 (24.8)	1273 (30.7)	<0.001	0.131	1167 (30.0)	1199 (30.8)	0.445	0.018
			1	11495 (75.2)	2879 (69.3)			2722 (70.0)	2690 (69.2)		
	Hypertension	I10–I15	0	6254 (40.9)	1945 (46.8)	<0.001	0.12	1804 (46.4)	1835 (47.2)	0.495	0.016
			1	9035 (59.1)	2207 (53.2)			2085 (53.6)	2054 (52.8)		
	Acute myocardial infarction	I21–I22	0	14910 (97.5)	3983 (95.9)	<0.001	0.09	3745 (96.3)	3742 (96.2)	0.905	0.004
			1	379 (2.5)	169 (4.1)			144 (3.7)	147 (3.8)		
	Cor pulmonale	I26–I27	0	15165 (99.2)	4082 (98.3)	<0.001	0.079	3839 (98.7)	3837 (98.7)	0.921	0.005
			1	124 (0.8)	70 (1.7)			50 (1.3)	52 (1.3)		
	Cor pulmonale and pulmonary circulation diseases,	I26–I28	0	15165 (99.2)	4082 (98.3)	<0.001	0.079	3839 (98.7)	3837 (98.7)	0.921	0.005

	including pulmonary embolism, pulmonary hypertension and cor pulmonale										
			1	124 (0.8)	70 (1.7)			50 (1.3)	52 (1.3)		
	Infarction and TIA	G45 , I63 , I66	0	11385 (74 .5)	3247 (78.2)	<0.001	0.088	3047 (78 .3)	3029 (77.9)	0.641	0.011
			1	3904 (25 .5)	905 (21.8)			842 (21 .7)	860 (22.1)		
	Acute upper respiratory tract infection	J00 - J06	0	14315 (93 .6)	3866 (93.1)	0.244	0.021	3625 (93 .2)	3625 (93.2)	1	<0.001
			1	974 (6.4)	286 (6.9)			264 (6.8)	264 (6.8)		
	asthma	J45 - J46	0	15043 (98 .4)	4082 (98.3)	0.781	0.006	3835 (98 .6)	3829 (98.5)	0.637	0.013
			1	246 (1.6)	70 (1.7)			54 (1.4)	60 (1.5)		
	respiratory failure	J80 , J96	0	15237 (99 .7)	4114 (99.1)	<0.001	0.073	3870 (99 .5)	3864 (99.4)	0.45	0.021
			1	52 (0.3)	38 (0.9)			19 (0.5)	25 (0.6)		
	Other respiratory diseases primarily affecting the interstitial tissue	J80 - J84	0	15172 (99 .2)	4086 (98.4)	<0.001	0.076	3853 (99 .1)	3840 (98.7)	0.191	0.032

			1	117 (0.8)	66 (1.6)			36 (0.9)	49 (1.3)		
	Digestive system diseases	K00–K93	0	10337 (67.6)	2579 (62.1)	<0.001	0.115	2516 (64.7)	2489 (64.0)	0.538	0.014
			1	4952 (32.4)	1573 (37.9)			1373 (35.3)	1400 (36.0)		
	Cirrhosis	K70, K74	0	15207 (99.5)	4093 (98.6)	<0.001	0.09	3854 (99.1)	3846 (98.9)	0.426	0.021
			1	82 (0.5)	59 (1.4)			35 (0.9)	43 (1.1)		
	Viral hepatitis	B15–B19	0	15134 (99.0)	4065 (97.9)	<0.001	0.088	3833 (98.6)	3823 (98.3)	0.411	0.021
			1	155 (1.0)	87 (2.1)			56 (1.4)	66 (1.7)		
	Pancreatic diseases	K85–K87	0	15228 (99.6)	4102 (98.8)	<0.001	0.09	3862 (99.3)	3859 (99.2)	0.79	0.009
			1	61 (0.4)	50 (1.2)			27 (0.7)	30 (0.8)		
	Other diseases of the digestive system	K90–K93	0	15122 (98.9)	4041 (97.3)	<0.001	0.117	3826 (98.4)	3824 (98.3)	0.929	0.004
			1	167 (1.1)	111 (2.7)			63 (1.6)	65 (1.7)		
	hepatitis	B15–B19, K73	0	15127 (98.9)	4060 (97.8)	<0.001	0.091	3830 (98.5)	3820 (98.2)	0.422	0.02
			1	162 (1.1)	92 (2.2)			59 (1.5)	69 (1.8)		
	arthritis	M00	0	1434	3897	0.9	0.	3665	3652	0.	0.0

		–M25		4 (93.8)	(93.9)	55	002	(94.2)	(93.9)	564	14
			1	945 (6.2)	255 (6.1)			224 (5.8)	237 (6.1)		
	Other back pain	M50–M54	0	14057 (91.9)	3857 (92.9)	0.046	0.036	3608 (92.8)	3611 (92.9)	0.93	0.003
			1	1232 (8.1)	295 (7.1)			281 (7.2)	278 (7.1)		
	Synovial and tendon disorders	M65–M68	0	15227 (99.6)	4140 (99.7)	0.348	0.02	3881 (99.8)	3877 (99.7)	0.502	0.02
			1	62 (0.4)	12 (0.3)			8 (0.2)	12 (0.3)		
	Disorders of bone density and structure	M80–M85	0	14923 (97.6)	3997 (96.3)	<0.001	0.078	3769 (96.9)	3771 (97.0)	0.948	0.003
			1	366 (2.4)	155 (3.7)			120 (3.1)	118 (3.0)		
	Fungal diseases	B35–B49	0	15200 (99.4)	4124 (99.3)	0.57	0.012	3869 (99.5)	3865 (99.4)	0.65	0.014
			1	89 (0.6)	28 (0.7)			20 (0.5)	24 (0.6)		
	Nephritis and kidney disease	N00–N19	0	14864 (97.2)	3939 (94.9)	<0.001	0.121	3740 (96.2)	3717 (95.6)	0.21	0.03
			1	425 (2.8)	213 (5.1)			149 (3.8)	172 (4.4)		
	Kidney disease	I12–I13, N03–	0	14956 (97.8)	3976 (95.8)	<0.001	0.117	3767 (96.9)	3750 (96.4)	0.314	0.024

		N04, N17-N19, R34									
			1	333 (2.2)	176 (4.2)			122 (3.1)	139 (3.6)		
	Chronic kidney disease	N18	0	15168 (99.2)	4081 (98.3)	<0.001	0.083	3842 (98.8)	3835 (98.6)	0.548	0.016
			1	121 (0.8)	71 (1.7)			47 (1.2)	54 (1.4)		
	Female genital conditions such as menstruation and menopause	N91-N95	0	15171 (99.2)	4102 (98.8)	0.01	0.044	3850 (99.0)	3849 (99.0)	1	0.003
			1	118 (0.8)	50 (1.2)			39 (1.0)	40 (1.0)		
	Pregnancy, childbirth and postpartum period	000-099	0	15181 (99.3)	4089 (98.5)	<0.001	0.077	3842 (98.8)	3842 (98.8)	1	<0.001
			1	108 (0.7)	63 (1.5)			47 (1.2)	47 (1.2)		
	Other maternal conditions primarily related to pregnancy	020-029	0	15234 (99.6)	4111 (99.0)	<0.001	0.077	3859 (99.2)	3861 (99.3)	0.895	0.006
			1	55 (0.4)	41 (1.0)			30 (0.8)	28 (0.7)		
	Symptoms and signs involving the	R00-R09	0	14390 (94.1)	3904 (94.0)	0.851	0.004	3673 (94.4)	3664 (94.2)	0.695	0.01

	circulatory and respiratory systems										
			1	899 (5.9)	248 (6.0)			216 (5.6)	225 (5.8)		
	Symptoms and signs involving cognition, perception, emotional state, and behavior	R40–R46	0	14581 (95.4)	3973 (95.7)	0.405	0.015	3727 (95.8)	3727 (95.8)	1	<0.001
			1	708 (4.6)	179 (4.3)			162 (4.2)	162 (4.2)		
	Abnormal blood test, no diagnosis	R70–R79	0	14961 (97.9)	4000 (96.3)	<0.001	0.09	3770 (96.9)	3765 (96.8)	0.794	0.007
			1	328 (2.1)	152 (3.7)			119 (3.1)	124 (3.2)		
	Abnormal findings on diagnostic imaging and functional studies, no diagnosis	R90–R94	0	14538 (95.1)	3867 (93.1)	<0.001	0.083	3658 (94.1)	3658 (94.1)	1	<0.001
			1	751 (4.9)	285 (6.9)			231 (5.9)	231 (5.9)		
	Hip and thigh injuries	S70–S79	0	15216 (99.5)	4121 (99.3)	0.047	0.035	3865 (99.4)	3865 (99.4)	1	<0.001
			1	73 (0.5)	31 (0.7)			24 (0.6)	24 (0.6)		
	Digestive system	C15–	0	15078	3988 (96.	<0.001	0.16	3801 (97	3802 (97.	1	0.002

	cancer	C26		(98.6)	1)			.7)	8)		
			1	211 (1.4)	164 (3.9)			88 (2.3)	87 (2.2)		
	Tumor	C00-C99, D00-D48	0	13806 (90.3)	3511 (84.6)	<0.001	0.174	3396 (87.3)	3388 (87.1)	0.812	0.006
			1	1483 (9.7)	641 (15.4)			493 (12.7)	501 (12.9)		
	Malignant tumors	C00-C98	0	14511 (94.9)	3726 (89.7)	<0.001	0.195	3591 (92.3)	3592 (92.4)	1	0.001
			1	778 (5.1)	426 (10.3)			298 (7.7)	297 (7.6)		
	Other malignant tumors	C00-C14, C17, C26-C31, C37-C39, C43-C49, C51-C52, C57-C60,	0	14881 (97.3)	3918 (94.4)	<0.001	0.149	3731 (95.9)	3734 (96.0)	0.908	0.004

		C62–C63, C68–C69, C73–C76, C80, C97									
			1	408 (2.7)	234 (5.6)			158 (4.1)	155 (4.0)		
	Urinary tract malignancy?	C64–C68	0	15233 (99.6)	4134 (99.6)	0.63	0.011	3871 (99.5)	3872 (99.6)	1	0.004
			1	56 (0.4)	18 (0.4)			18 (0.5)	17 (0.4)		
	Malignant tumors of endocrine glands	C73–C75	0	15219 (99.5)	4131 (99.5)	0.785	0.007	3868 (99.5)	3871 (99.5)	0.748	0.011
			1	70 (0.5)	21 (0.5)			21 (0.5)	18 (0.5)		
	Uterine malignancy	C53–C55	0	15247 (99.7)	4123 (99.3)	<0.001	0.061	3873 (99.6)	3870 (99.5)	0.735	0.012
			1	42 (0.3)	29 (0.7)			16 (0.4)	19 (0.5)		
	Lung diseases due to foreign substances	J60–J70	0	15264 (99.8)	4136 (99.6)	0.01	0.042	3878 (99.7)	3875 (99.6)	0.689	0.014
			1	25 (0.2)	16 (0.4)			11 (0.3)	14 (0.4)		
	Other	J95	0	1469	3906	<0.	0.	3709	3691	0.	0.0

	diseases of the respiratory system	-J99		4 (96.1)	(94.1)	001	094	(95.4)	(94.9)	37	22
			1	595 (3.9)	246 (5.9)			180 (4.6)	198 (5.1)		
	anemia	D50-D64	0	14921 (97.6)	3935 (94.8)	<0.001	0.148	3741 (96.2)	3736 (96.1)	0.814	0.007
			1	368 (2.4)	217 (5.2)			148 (3.8)	153 (3.9)		
	Diseases of the blood and blood-forming organs and certain diseases involving the immune system	D50-D89	0	14520 (95.0)	3805 (91.6)	<0.001	0.133	3628 (93.3)	3624 (93.2)	0.892	0.004
			1	769 (5.0)	347 (8.4)			261 (6.7)	265 (6.8)		
	Bone disease and osteomalacia	M80-M94	0	14802 (96.8)	3960 (95.4)	<0.001	0.074	3728 (95.9)	3736 (96.1)	0.687	0.01
			1	487 (3.2)	192 (4.6)			161 (4.1)	153 (3.9)		
	Kidney and bladder diseases	N23-N29, N31-N33, N35-N39,	0	13735 (89.8)	3614 (87.0)	<0.001	0.087	3436 (88.4)	3419 (87.9)	0.575	0.014

		N41-N64									
			1	1554 (10.2)	538 (13.0)			453 (11.6)	470 (12.1)		
	Nutritional disorders other than lipidemia	E40-E77, E79-E90	0	14446 (94.5)	3808 (91.7)	<0.001	0.11	3628 (93.3)	3616 (93.0)	0.622	0.012
			1	843 (5.5)	344 (8.3)			261 (6.7)	273 (7.0)		
	Other nutritional deficiencies	E50-E64	0	15254 (99.8)	4138 (99.7)	0.289	0.02	3879 (99.7)	3878 (99.7)	1	0.005
			1	35 (0.2)	14 (0.3)			10 (0.3)	11 (0.3)		
	Obesity and other overnutrition	E65-E68	0	15222 (99.6)	4105 (98.9)	<0.001	0.079	3862 (99.3)	3858 (99.2)	0.693	0.012
			1	67 (0.4)	47 (1.1)			27 (0.7)	31 (0.8)		
	Metabolic disorders	E70-E90	0	12776 (83.6)	3314 (79.8)	<0.001	0.097	3186 (81.9)	3150 (81.0)	0.307	0.024
			1	2513 (16.4)	838 (20.2)			703 (18.1)	739 (19.0)		
	Neurotic, stress-related and somatoform disorders	F40-F48	0	14437 (94.4)	3827 (92.2)	<0.001	0.09	3627 (93.3)	3604 (92.7)	0.329	0.023
			1	852 (5.6)	325 (7.8)			262 (6.7)	285 (7.3)		
	Penicillin V		0	1522	4106	<0.	0.	3853	3849	0.	0.0

	Potassium			6 (99.6)	(98.9)	001	08	(99.1)	(99.0)	729	1
			1	63 (0.4)	46 (1.1)			36 (0.9)	40 (1.0)		
	Cefixime		0	15245 (99.7)	4150 (100.0)	0.008	0.059	3887 (99.9)	3887 (99.9)	1	<0.001
			1	44 (0.3)	2 (0.0)			2 (0.1)	2 (0.1)		
	Cefuroxime		0	15194 (99.4)	4139 (99.7)	0.024	0.045	3877 (99.7)	3876 (99.7)	1	0.005
			1	95 (0.6)	13 (0.3)			12 (0.3)	13 (0.3)		
	Vitamin B1		0	15113 (98.8)	4135 (99.6)	<0.001	0.084	3878 (99.7)	3874 (99.6)	0.556	0.018
			1	176 (1.2)	17 (0.4)			11 (0.3)	15 (0.4)		
	Vitamin C		0	15014 (98.2)	4040 (97.3)	<0.001	0.061	3812 (98.0)	3803 (97.8)	0.527	0.016
			1	275 (1.8)	112 (2.7)			77 (2.0)	86 (2.2)		
	glucose		0	15120 (98.9)	4077 (98.2)	<0.001	0.059	3836 (98.6)	3829 (98.5)	0.57	0.015
			1	169 (1.1)	75 (1.8)			53 (1.4)	60 (1.5)		
	Potassium chloride		0	14825 (97.0)	3956 (95.3)	<0.001	0.087	3748 (96.4)	3752 (96.5)	0.855	0.006
			1	464 (3.0)	196 (4.7)			141 (3.6)	137 (3.5)		

	Sodium chloride	0	9480 (62.0)	2601 (62.6)	0.462	0.013	2503 (64.4)	2504 (64.4)	1	0.001
		1	5809 (38.0)	1551 (37.4)			1386 (35.6)	1385 (35.6)		
	Water for injection	0	15186 (99.3)	4106 (98.9)	0.006	0.046	3867 (99.4)	3863 (99.3)	0.664	0.013
		1	103 (0.7)	46 (1.1)			22 (0.6)	26 (0.7)		
	Levothyroxine	0	15192 (99.4)	4141 (99.7)	0.006	0.055	3878 (99.7)	3878 (99.7)	1	<0.001
		1	97 (0.6)	11 (0.3)			11 (0.3)	11 (0.3)		
	Oxaliplatin	0	15260 (99.8)	4148 (99.9)	0.279	0.025	3886 (99.9)	3885 (99.9)	1	0.009
		1	29 (0.2)	4 (0.1)			3 (0.1)	4 (0.1)		
	Edaravone	0	15214 (99.5)	4127 (99.4)	0.442	0.015	3867 (99.4)	3865 (99.4)	0.882	0.007
		1	75 (0.5)	25 (0.6)			22 (0.6)	24 (0.6)		
	Aspirin clears heat	0	14419 (94.3)	4005 (96.5)	<0.001	0.103	3739 (96.1)	3749 (96.4)	0.59	0.014
		1	870 (5.7)	147 (3.5)			150 (3.9)	140 (3.6)		
	Compound paracetamol	0	15122 (98.9)	4128 (99.4)	0.004	0.057	3865 (99.4)	3865 (99.4)	1	<0.001
		1	167 (1.1)	24 (0.6)			24 (0.6)	24 (0.6)		

	Isosorbide dinitrate	0	15089 (98.7)	4093 (98.6)	0.627	0.01	3843 (98.8)	3842 (98.8)	1	0.002
		1	200 (1.3)	59 (1.4)			46 (1.2)	47 (1.2)		
	Felodipine	0	14948 (97.8)	4117 (99.2)	<0.001	0.113	3859 (99.2)	3856 (99.2)	0.8	0.009
		1	341 (2.2)	35 (0.8)			30 (0.8)	33 (0.8)		
	Nifedipine	0	13249 (86.7)	3828 (92.2)	<0.001	0.181	3584 (92.2)	3583 (92.1)	1	0.001
		1	2040 (13.3)	324 (7.8)			305 (7.8)	306 (7.9)		
	Valsartan	0	13464 (88.1)	3852 (92.8)	<0.001	0.161	3592 (92.4)	3605 (92.7)	0.605	0.013
		1	1825 (11.9)	300 (7.2)			297 (7.6)	284 (7.3)		
	Fenofibrate	0	15131 (99.0)	4139 (99.7)	<0.001	0.088	3874 (99.6)	3877 (99.7)	0.7	0.013
		1	158 (1.0)	13 (0.3)			15 (0.4)	12 (0.3)		
	aspirin	0	10892 (71.2)	3486 (84.0)	<0.001	0.309	3254 (83.7)	3250 (83.6)	0.927	0.003
		1	4397 (28.8)	666 (16.0)			635 (16.3)	639 (16.4)		
	Pantoprazole	0	14900 (97.5)	3975 (95.7)	<0.001	0.095	3768 (96.9)	3766 (96.8)	0.948	0.003
		1	389 (2.	177 (4.3			121 (3.	123 (3.2		

				5)	)			1)	)		
	Torsemide		0	15212 (99.5)	4112 (99.0)	0.001	0.054	3866 (99.4)	3864 (99.4)	0.885	0.007
			1	77 (0.5)	40 (1.0)			23 (0.6)	25 (0.6)		
	Furosemide		0	14915 (97.6)	4029 (97.0)	0.07	0.032	3810 (98.0)	3796 (97.6)	0.316	0.024
			1	374 (2.4)	123 (3.0)			79 (2.0)	93 (2.4)		
	Hemocoagulase		0	15267 (99.9)	4136 (99.6)	0.003	0.047	3884 (99.9)	3882 (99.8)	0.773	0.013
			1	22 (0.1)	16 (0.4)			5 (0.1)	7 (0.2)		
	Low molecular weight heparin calcium		0	15038 (98.4)	4055 (97.7)	0.003	0.05	3819 (98.2)	3813 (98.0)	0.676	0.011
			1	251 (1.6)	97 (2.3)			70 (1.8)	76 (2.0)		
	Cold and heat		0	15205 (99.5)	4145 (99.8)	0.002	0.064	3882 (99.8)	3882 (99.8)	1	<0.001
			1	84 (0.5)	7 (0.2)			7 (0.2)	7 (0.2)		
	Cold Remedy		0	15236 (99.7)	4149 (99.9)	0.006	0.06	3883 (99.8)	3886 (99.9)	0.505	0.023
			1	53 (0.3)	3 (0.1)			6 (0.2)	3 (0.1)		
	Anti-inflammatory and choleretic		0	15264 (99.8)	4151 (100.0)	0.052	0.046	3888 (100.0)	3888 (100.0)	1	<0.001
			1	25	1			1	1		

				(0.2)	(0.0)			(0.0)	(0.0)		
	Xinhuang		0	15222 (99.6)	4145 (99.8)	0.018	0.049	3883 (99.8)	3882 (99.8)	1	0.006
			1	67 (0.4)	7 (0.2)			6 (0.2)	7 (0.2)		
	Ginkgo biloba Chinese medicine		0	14688 (96.1)	4067 (98.0)	<0.001	0.111	3809 (97.9)	3807 (97.9)	0.937	0.004
			1	601 (3.9)	85 (2.0)			80 (2.1)	82 (2.1)		
	Compound Danshen		0	14321 (93.7)	4015 (96.7)	<0.001	0.142	3757 (96.6)	3759 (96.7)	0.95	0.003
			1	968 (6.3)	137 (3.3)			132 (3.4)	130 (3.3)		
	Safflower yellow		0	15204 (99.4)	4126 (99.4)	0.677	0.009	3866 (99.4)	3869 (99.5)	0.76	0.01
			1	85 (0.6)	26 (0.6)			23 (0.6)	20 (0.5)		
	Activate blood circulation and relieve pain		0	15242 (99.7)	4149 (99.9)	0.013	0.054	3885 (99.9)	3886 (99.9)	1	0.009
			1	47 (0.3)	3 (0.1)			4 (0.1)	3 (0.1)		
	Salvia miltiorrhiza		0	14982 (98.0)	4107 (98.9)	<0.001	0.075	3846 (98.9)	3847 (98.9)	1	0.002
			1	307 (2.0)	45 (1.1)			43 (1.1)	42 (1.1)		
	Vitreous surgery		0	15272 (99.9)	4143 (99.8)	0.158	0.026	3887 (99.9)	3886 (99.9)	1	0.01

			1	17 (0.1)	9 (0.2)			2 (0.1)	3 (0.1)		
	Diagnostic procedures of the stomach		0	15252 (99.8)	4124 (99.3)	<0.001	0.064	3870 (99.5)	3868 (99.5)	0.874	0.007
			1	37 (0.2)	28 (0.7)			19 (0.5)	21 (0.5)		
	Soft tissue x-rays of the face, head, and neck		0	15232 (99.6)	4119 (99.2)	0.001	0.055	3864 (99.4)	3865 (99.4)	1	0.003
			1	57 (0.4)	33 (0.8)			25 (0.6)	24 (0.6)		
	Abdominal soft tissue X-ray examination		0	15263 (99.8)	4132 (99.5)	<0.001	0.055	3879 (99.7)	3878 (99.7)	1	0.005
			1	26 (0.2)	20 (0.5)			10 (0.3)	11 (0.3)		

Heart failure

outcome	Baseline variables	ICD-10	level	before matching				after matching			
				Single drug[a]	Strengthen	p	SMD	Single drug[a]	Strengthen	p	SMD
heart failure	Paroxysmal and episodic disorders	G40-G47	0	13557 (89.1)	3653 (90.6)	0.006	0.05	3434 (90.8)	3426 (90.6)	0.781	0.007
			1	1652 (10.9)	377 (9.4)			347 (9.2)	355 (9.4)		
	Nerve: Nerve root and plexus disorders	G50-G59	0	14963 (98.4)	3985 (98.9)	0.025	0.043	3740 (98.9)	3739 (98.9)	1	0.003
			1	246 (1.	45 (1.1)			41 (1.	42 (1.1)		

				6)				1)			
	Conjunctival diseases	H10 - H13	0	14952 (98.3)	3950 (98.0)	0.229	0.022	3713 (98.2)	3714 (98.2)	1	0.002
			1	257 (1.7)	80 (2.0)			68 (1.8)	67 (1.8)		
	Lens diseases	H25 - H28	0	14179 (93.2)	3714 (92.2)	0.02	0.041	3499 (92.5)	3501 (92.6)	0.965	0.002
			1	1030 (6.8)	316 (7.8)			282 (7.5)	280 (7.4)		
	Sensory disorders (ear)	H60 - H95	0	14783 (97.2)	3916 (97.2)	0.967	0.002	3682 (97.4)	3681 (97.4)	1	0.002
			1	426 (2.8)	114 (2.8)			99 (2.6)	100 (2.6)		
	Cardiovascular disease	I00 - I15, I20 - I28, I30 - I51, I60 - I79	0	3784 (24.9)	1264 (31.4)	<0.001	0.145	1188 (31.4)	1195 (31.6)	0.882	0.004
			1	11425 (75.1)	2766 (68.6)			2593 (68.6)	2586 (68.4)		
	Hypertension	I10 - I15	0	6233 (41.0)	1914 (47.5)	<0.001	0.131	1822 (48.2)	1806 (47.8)	0.73	0.008
			1	8976 (59	2116 (52.5)			1959 (51	1975 (52.2)		

				.0)				.8)			
	Acute myocardial infarction	I21 - I22	0	14861 (97.7)	3898 (96.7)	<0.001	0.06	3663 (96.9)	3665 (96.9)	0.947	0.003
			1	348 (2.3)	132 (3.3)			118 (3.1)	116 (3.1)		
	Cor pulmonale	I26 - I27	0	15101 (99.3)	3976 (98.7)	<0.001	0.063	3742 (99.0)	3739 (98.9)	0.823	0.008
			1	108 (0.7)	54 (1.3)			39 (1.0)	42 (1.1)		
	Cor pulmonale and pulmonary circulation diseases, including pulmonary embolism, pulmonary hypertension and cor pulmonale	I26 - I28	0	15101 (99.3)	3976 (98.7)	<0.001	0.063	3742 (99.0)	3739 (98.9)	0.823	0.008
			1	108 (0.7)	54 (1.3)			39 (1.0)	42 (1.1)		
	Ischemic stroke	I63 - I64	0	12057 (79.3)	3281 (81.4)	0.003	0.054	3089 (81.7)	3075 (81.3)	0.7	0.01
			1	3152 (20.7)	749 (18.6)			692 (18.3)	706 (18.7)		
	Varicose veins, hemorrhoids	I83 - I86	0	14888 (97.9)	3943 (97.8)	0.899	0.003	3707 (98.0)	3709 (98.1)	0.933	0.004
			1	321 (2.1)	87 (2.2)			74 (2.0)	72 (1.9)		

	Acute upper respiratory tract infection	J00-J06	0	14245 (93.7)	3764 (93.4)	0.57	0.011	3545 (93.8)	3535 (93.5)	0.672	0.011
			1	964 (6.3)	266 (6.6)			236 (6.2)	246 (6.5)		
	respiratory failure	J80, J96	0	15163 (99.7)	4001 (99.3)	<0.001	0.059	3764 (99.6)	3762 (99.5)	0.867	0.008
			1	46 (0.3)	29 (0.7)			17 (0.4)	19 (0.5)		
	Other respiratory diseases primarily affecting the interstitial tissue	J80-J84	0	15096 (99.3)	3972 (98.6)	<0.001	0.067	3741 (98.9)	3739 (98.9)	0.912	0.005
			1	113 (0.7)	58 (1.4)			40 (1.1)	42 (1.1)		
	Digestive system diseases	K00-K93	0	10321 (67.9)	2513 (62.4)	<0.001	0.116	2456 (65.0)	2435 (64.4)	0.63	0.012
			1	4888 (32.1)	1517 (37.6)			1325 (35.0)	1346 (35.6)		
	Pancreatic diseases	K85-K87	0	15150 (99.6)	3984 (98.9)	<0.001	0.087	3759 (99.4)	3756 (99.3)	0.77	0.01
			1	59 (0.4)	46 (1.1)			22 (0.6)	25 (0.7)		
	Other diseases of the digestive system	K90-K93	0	15046 (98.9)	3926 (97.4)	<0.001	0.113	3718 (98.3)	3719 (98.4)	1	0.002
			1	163 (1.1)	104 (2.6)			63 (1.7)	62 (1.6)		

	hepatitis	B15-B19, K73	0	15049 (98.9)	3939 (97.7)	<0.001	0.095	3723 (98.5)	3720 (98.4)	0.853	0.006
			1	160 (1.1)	91 (2.3)			58 (1.5)	61 (1.6)		
	arthritis	M00-M25	0	14276 (93.9)	3792 (94.1)	0.615	0.01	3555 (94.0)	3557 (94.1)	0.961	0.002
			1	933 (6.1)	238 (5.9)			226 (6.0)	224 (5.9)		
	Other back pain	M50-M54	0	13985 (92.0)	3752 (93.1)	0.017	0.044	3525 (93.2)	3524 (93.2)	1	0.001
			1	1224 (8.0)	278 (6.9)			256 (6.8)	257 (6.8)		
	Synovial and tendon disorders	M65-M68	0	15147 (99.6)	4019 (99.7)	0.275	0.023	3774 (99.8)	3772 (99.8)	0.802	0.012
			1	62 (0.4)	11 (0.3)			7 (0.2)	9 (0.2)		
	Disorders of bone density and structure	M80-M85	0	14848 (97.6)	3876 (96.2)	<0.001	0.084	3678 (97.3)	3659 (96.8)	0.223	0.03
			1	361 (2.4)	154 (3.8)			103 (2.7)	122 (3.2)		
	Nephritis	N00-N01, N03-N05, N0	0	14958 (98.3)	3897 (96.7)	<0.001	0.106	3679 (97.3)	3678 (97.3)	1	0.002

		8, N10-N12, N15-N16, N18									
			1	251 (1.7)	133 (3.3)			102 (2.7)	103 (2.7)		
	Fungal diseases	B35-B49	0	15123 (99.4)	4005 (99.4)	0.77	0.007	3762 (99.5)	3761 (99.5)	1	0.004
			1	86 (0.6)	25 (0.6)			19 (0.5)	20 (0.5)		
	Nephritis and kidney disease	N00-N19	0	14803 (97.3)	3833 (95.1)	<0.001	0.117	3633 (96.1)	3620 (95.7)	0.486	0.017
			1	406 (2.7)	197 (4.9)			148 (3.9)	161 (4.3)		
	Kidney disease	N00-N29	0	13904 (91.4)	3568 (88.5)	<0.001	0.096	3404 (90.0)	3385 (89.5)	0.494	0.017
			1	1305 (8.6)	462 (11.5)			377 (10.0)	396 (10.5)		
	Kidney disease	I12-I13, N03-N04, N17-N1	0	14894 (97.9)	3867 (96.0)	<0.001	0.115	3660 (96.8)	3653 (96.6)	0.699	0.01

		9, R34									
			1	315 (2.1)	163 (4.0)			121 (3.2)	128 (3.4)		
	Chronic kidney disease	N18	0	15097 (99.3)	3967 (98.4)	<0.001	0.078	3739 (98.9)	3735 (98.8)	0.748	0.01
			1	112 (0.7)	63 (1.6)			42 (1.1)	46 (1.2)		
	Other genitourinary diseases	N23-N29, N31-N33, N35-N39, N41-N60, N62-N64	0	13666 (89.9)	3522 (87.4)	<0.001	0.078	3366 (89.0)	3336 (88.2)	0.294	0.025
			1	1543 (10.1)	508 (12.6)			415 (11.0)	445 (11.8)		
	Pregnancy, childbirth and postpartum period	O00-O99	0	15107 (99.3)	3968 (98.5)	<0.001	0.083	3733 (98.7)	3731 (98.7)	0.919	0.005
			1	102 (0.7)	62 (1.5)			48 (1.3)	50 (1.3)		
	Symptoms and signs	R00-	0	14332	3810 (94.5)	0.478	0.013	3597	3581 (94.7)	0.432	0.019

	involving the circulatory and respiratory systems	R09		(94.2)				(95.1)			
			1	877 (5.8)	220 (5.5)			184 (4.9)	200 (5.3)		
	Symptoms and signs involving cognition, perception, emotional state, and behavior	R40 - R46	0	14483 (95.2)	3863 (95.9)	0.1	0.031	3625 (95.9)	3628 (96.0)	0.908	0.004
			1	726 (4.8)	167 (4.1)			156 (4.1)	153 (4.0)		
	Abnormal blood test, no diagnosis	R70 - R79	0	14889 (97.9)	3882 (96.3)	<0.001	0.094	3663 (96.9)	3657 (96.7)	0.744	0.009
			1	320 (2.1)	148 (3.7)			118 (3.1)	124 (3.3)		
	Abnormal findings on diagnostic imaging and functional studies, no diagnosis	R90 - R94	0	14467 (95.1)	3769 (93.5)	<0.001	0.069	3560 (94.2)	3569 (94.4)	0.692	0.01
			1	742 (4.9)	261 (6.5)			221 (5.8)	212 (5.6)		
	Digestive system cancer	C15 - C26	0	15005 (98.7)	3871 (96.1)	<0.001	0.163	3697 (97.8)	3689 (97.6)	0.593	0.014
			1	204 (1.3)	159 (3.9)			84 (2.2)	92 (2.4)		
	Surgical and medical complication	T80 - T88	0	15159 (99	4003 (99.3)	0.004	0.048	3763 (99	3758 (99.4)	0.531	0.018

	s, not otherwise classified			.7)				.5)			
			1	50 (0.3)	27 (0.7)			18 (0.5)	23 (0.6)		
	Tumor	C00-C99, D00-D48	0	13755 (90.4)	3409 (84.6)	<0.001	0.178	3319 (87.8)	3283 (86.8)	0.227	0.029
			1	1454 (9.6)	621 (15.4)			462 (12.2)	498 (13.2)		
	Malignant tumors	C00-C98	0	14454 (95.0)	3620 (89.8)	<0.001	0.198	3507 (92.8)	3484 (92.1)	0.338	0.023
			1	755 (5.0)	410 (10.2)			274 (7.2)	297 (7.9)		
	Other malignant tumors	C00-C14, C17, C26-C31, C37-C39, C43-C49, C51-C52,	0	14815 (97.4)	3804 (94.4)	<0.001	0.153	3643 (96.4)	3624 (95.8)	0.285	0.026

		C57-C60, C62-C63, C68-C69, C73-C76, C80, C97									
			1	394 (2.6)	226 (5.6)			138 (3.6)	157 (4.2)		
	Malignant tumors of endocrine glands	C73-C75	0	15142 (99.6)	4010 (99.5)	0.736	0.008	3765 (99.6)	3762 (99.5)	0.735	0.012
			1	67 (0.4)	20 (0.5)			16 (0.4)	19 (0.5)		
	Other anemia	D62-D64	0	14930 (98.2)	3873 (96.1)	<0.001	0.124	3677 (97.2)	3671 (97.1)	0.729	0.01
			1	279 (1.8)	157 (3.9)			104 (2.8)	110 (2.9)		
	Uterine malignancy	C53-C55	0	15169 (99.7)	4001 (99.3)	<0.001	0.065	3765 (99.6)	3762 (99.5)	0.735	0.012
			1	40 (0.3)	29 (0.7)			16 (0.4)	19 (0.5)		
	Lung diseases due to foreign	J60-J70	0	15183 (99	4012 (99.6)	0.002	0.05	3769 (99	3766 (99.6)	0.7	0.013

	substances			.8)				.7)			
			1	26 (0.2)	18 (0.4)			12 (0.3)	15 (0.4)		
	Other diseases of the respiratory system	J95-J99	0	14647 (96.3)	3813 (94.6)	<0.001	0.081	3610 (95.5)	3601 (95.2)	0.662	0.011
			1	562 (3.7)	217 (5.4)			171 (4.5)	180 (4.8)		
	anemia	D50-D64	0	14856 (97.7)	3840 (95.3)	<0.001	0.13	3651 (96.6)	3640 (96.3)	0.536	0.016
			1	353 (2.3)	190 (4.7)			130 (3.4)	141 (3.7)		
	Diseases of the blood and blood-forming organs and certain diseases involving the immune system	D50-D89	0	14465 (95.1)	3711 (92.1)	<0.001	0.124	3544 (93.7)	3529 (93.3)	0.513	0.016
			1	744 (4.9)	319 (7.9)			237 (6.3)	252 (6.7)		
	Bone disease and osteomalacia	M80-M94	0	14730 (96.9)	3840 (95.3)	<0.001	0.081	3641 (96.3)	3623 (95.8)	0.315	0.024
			1	479 (3.1)	190 (4.7)			140 (3.7)	158 (4.2)		
	Anemia or coagulation disorders	D60-D69	0	14841 (97.6)	3836 (95.2)	<0.001	0.129	3648 (96.5)	3639 (96.2)	0.623	0.013
			1	368 (2.4)	194 (4.8)			133 (3.5)	142 (3.8)		

	Kidney and bladder diseases	N23-N29, N31-N33, N35-N39, N41-N64	0	13662 (89.8)	3520 (87.3)	<0.001	0.078	3362 (88.9)	3334 (88.2)	0.33	0.023
			1	1547 (10.2)	510 (12.7)			419 (11.1)	447 (11.8)		
	diabetes	E10-E14	0	710 (4.7)	321 (8.0)	<0.001	0.136	339 (9.0)	307 (8.1)	0.202	0.03
			1	14499 (95.3)	3709 (92.0)			3442 (91.0)	3474 (91.9)		
	Nutritional disorders other than lipidemia	E40-E77, E79-E90	0	14371 (94.5)	3705 (91.9)	<0.001	0.102	3539 (93.6)	3522 (93.1)	0.459	0.018
			1	838 (5.5)	325 (8.1)			242 (6.4)	259 (6.9)		
	Obesity and other overnutrition	E65-E68	0	15145 (99.6)	3980 (98.8)	<0.001	0.09	3752 (99.2)	3754 (99.3)	0.893	0.006
			1	64 (0.4)	50 (1.2)			29 (0.8)	27 (0.7)		
	Metabolic disorders	E70-	0	12721	3236 (80.3)	<0.001	0.087	3113	3070 (81.2)	0.211	0.029

		E90		(83.6)				(82.3)			
			1	2488 (16.4)	794 (19.7)			668 (17.7)	711 (18.8)		
	Neurotic , stress-related and somatoform disorders	F40 - F48	0	14378 (94.5)	3728 (92.5)	<0.001	0.083	3540 (93.6)	3514 (92.9)	0.251	0.027
			1	831 (5.5)	302 (7.5)			241 (6.4)	267 (7.1)		
	Penicillin V Potassium		0	15145 (99.6)	3985 (98.9)	<0.001	0.08	3745 (99.0)	3739 (98.9)	0.569	0.016
			1	64 (0.4)	45 (1.1)			36 (1.0)	42 (1.1)		
	Cefixime		0	15166 (99.7)	4030 (100.0)	0.001	0.075	3781 (100.0)	3781 (100.0)		<0.001
			1	43 (0.3)	0 (0.0)			3770 (99.7)	3772 (99.8)	0.823	0.01
	Cefuroxime		0	15116 (99.4)	4021 (99.8)	0.004	0.06	3772 (99.8)	3772 (99.8)	1	<0.001
			1	93 (0.6)	9 (0.2)			9 (0.2)	9 (0.2)		
	Vitamin B1		0	15030 (98.8)	4013 (99.6)	<0.001	0.085	3764 (99.6)	3765 (99.6)	1	0.004
			1	179 (1.2)	17 (0.4)			17 (0.4)	16 (0.4)		
	Vitamin C		0	14942 (98	3922 (97.3)	<0.001	0.063	3699 (97	3703 (97.9)	0.811	0.007

				.2)				.8)			
			1	267 (1.8)	108 (2.7)			82 (2.2)	78 (2.1)		
	glucose		0	15042 (98.9)	3959 (98.2)	0.001	0.056	3730 (98.7)	3727 (98.6)	0.844	0.007
			1	167 (1.1)	71 (1.8)			51 (1.3)	54 (1.4)		
	Potassium chloride		0	14761 (97.1)	3859 (95.8)	<0.001	0.07	3661 (96.8)	3658 (96.7)	0.896	0.004
			1	448 (2.9)	171 (4.2)			120 (3.2)	123 (3.3)		
	Sodium chloride		0	9464 (62.2)	2565 (63.6)	0.101	0.029	2451 (64.8)	2466 (65.2)	0.736	0.008
			1	5745 (37.8)	1465 (36.4)			1330 (35.2)	1315 (34.8)		
	Water for injection		0	15106 (99.3)	3988 (99.0)	0.023	0.04	3757 (99.4)	3759 (99.4)	0.882	0.007
			1	103 (0.7)	42 (1.0)			24 (0.6)	22 (0.6)		
	Levothyroxine		0	15114 (99.4)	4019 (99.7)	0.01	0.053	3770 (99.7)	3770 (99.7)	1	<0.001
			1	95 (0.6)	11 (0.3)			11 (0.3)	11 (0.3)		
	Oxaliplatin		0	15180 (99.8)	4026 (99.9)	0.302	0.024	3777 (99.9)	3777 (99.9)	1	<0.001
			1	29 (0.	4 (0.1)			4 (0.	4 (0.1)		

				2)				1)			
	Edaravone		0	15138 (99.5)	4007 (99.4)	0.475	0.014	3762 (99.5)	3759 (99.4)	0.754	0.011
			1	71 (0.5)	23 (0.6)			19 (0.5)	22 (0.6)		
	Aspirin clears heat		0	14345 (94.3)	3899 (96.7)	<0.001	0.118	3656 (96.7)	3656 (96.7)	1	<0.001
			1	864 (5.7)	131 (3.3)			125 (3.3)	125 (3.3)		
	Terbutaline		0	15183 (99.8)	4016 (99.7)	0.046	0.035	3774 (99.8)	3774 (99.8)	1	<0.001
			1	26 (0.2)	14 (0.3)			7 (0.2)	7 (0.2)		
	Compound paracetamol		0	15042 (98.9)	4007 (99.4)	0.003	0.058	3756 (99.3)	3758 (99.4)	0.885	0.007
			1	167 (1.1)	23 (0.6)			25 (0.7)	23 (0.6)		
	Isosorbide mononitrate		0	13664 (89.8)	3782 (93.8)	<0.001	0.147	3536 (93.5)	3550 (93.9)	0.538	0.015
			1	1545 (10.2)	248 (6.2)			245 (6.5)	231 (6.1)		
	Isosorbide dinitrate		0	15019 (98.8)	3980 (98.8)	1	0.001	3742 (99.0)	3744 (99.0)	0.908	0.005
			1	190 (1.2)	50 (1.2)			39 (1.0)	37 (1.0)		
	Bisoprolol		0	14967	4004 (99.4)	<0.001	0.09	3759	3756 (99.3)	0.77	0.01

				(98.4)				(99.4)			
			1	242 (1.6)	26 (0.6)			22 (0.6)	25 (0.7)		
	Felodipine		0	14868 (97.8)	3997 (99.2)	<0.001	0.116	3748 (99.1)	3749 (99.2)	1	0.003
			1	341 (2.2)	33 (0.8)			33 (0.9)	32 (0.8)		
	Nifedipine		0	13188 (86.7)	3718 (92.3)	<0.001	0.182	3496 (92.5)	3485 (92.2)	0.666	0.011
			1	2021 (13.3)	312 (7.7)			285 (7.5)	296 (7.8)		
	Fenofibrate		0	15052 (99.0)	4017 (99.7)	<0.001	0.087	3767 (99.6)	3769 (99.7)	0.844	0.009
			1	157 (1.0)	13 (0.3)			14 (0.4)	12 (0.3)		
	aspirin		0	10870 (71.5)	3409 (84.6)	<0.001	0.321	3185 (84.2)	3190 (84.4)	0.899	0.004
			1	4339 (28.5)	621 (15.4)			596 (15.8)	591 (15.6)		
	Pantoprazole		0	14834 (97.5)	3873 (96.1)	<0.001	0.082	3669 (97.0)	3670 (97.1)	1	0.002
			1	375 (2.5)	157 (3.9)			112 (3.0)	111 (2.9)		
	Torsemide		0	15137 (99.5)	3993 (99.1)	0.001	0.054	3760 (99.4)	3762 (99.5)	0.874	0.007

		1	72 (0.5)	37 (0.9)			21 (0.6)	19 (0.5)		
	Hemocoagulase	0	15189 (99.9)	4014 (99.6)	0.001	0.052	3773 (99.8)	3775 (99.8)	0.789	0.012
		1	20 (0.1)	16 (0.4)			8 (0.2)	6 (0.2)		
	Low molecular weight heparin calcium	0	14975 (98.5)	3940 (97.8)	0.003	0.051	3713 (98.2)	3709 (98.1)	0.798	0.008
		1	234 (1.5)	90 (2.2)			68 (1.8)	72 (1.9)		
	Cold and heat	0	15125 (99.4)	4025 (99.9)	0.001	0.074	3775 (99.8)	3777 (99.9)	0.752	0.015
		1	84 (0.6)	5 (0.1)			6 (0.2)	4 (0.1)		
	Cold Remedy	0	15159 (99.7)	4027 (99.9)	0.01	0.057	3776 (99.9)	3778 (99.9)	0.724	0.016
		1	50 (0.3)	3 (0.1)			5 (0.1)	3 (0.1)		
	Sanguisorba officinalis	0	15195 (99.9)	4018 (99.7)	0.004	0.047	3775 (99.8)	3775 (99.8)	1	<0.001
		1	14 (0.1)	12 (0.3)			6 (0.2)	6 (0.2)		
	Compound Danshen	0	14254 (93.7)	3910 (97.0)	<0.001	0.158	3668 (97.0)	3664 (96.9)	0.841	0.006
		1	955 (6.3)	120 (3.0)			113 (3.0)	117 (3.1)		
	Activate blood	0	151	4028	0.00	0.0	377	3779	0.6	0.01

	circulation and relieve pain			64 (99.7)	(100.0)	8	59	7 (99.9)	(99.9)	83	9
			1	45 (0.3)	2 (0.0)			4 (0.1)	2 (0.1)		
	Salvia miltiorrhiza		0	14906 (98.0)	3989 (99.0)	<0.001	0.08	3745 (99.0)	3742 (99.0)	0.816	0.008
			1	303 (2.0)	41 (1.0)			36 (1.0)	39 (1.0)		
	Intraocular lens implantation		0	15174 (99.8)	4009 (99.5)	0.004	0.048	3762 (99.5)	3766 (99.6)	0.606	0.016
			1	35 (0.2)	21 (0.5)			19 (0.5)	15 (0.4)		
	Diagnostic procedures of the stomach		0	15171 (99.8)	4002 (99.3)	<0.001	0.065	3761 (99.5)	3760 (99.4)	1	0.004
			1	38 (0.2)	28 (0.7)			20 (0.5)	21 (0.6)		
	Soft tissue x-rays of the face, head, and neck		0	15151 (99.6)	4002 (99.3)	0.012	0.043	3758 (99.4)	3757 (99.4)	1	0.003
			1	58 (0.4)	28 (0.7)			23 (0.6)	24 (0.6)		
	Abdominal soft tissue X-ray examination		0	15181 (99.8)	4009 (99.5)	<0.001	0.057	3771 (99.7)	3772 (99.8)	1	0.005
			1	28 (0.2)	21 (0.5)			10 (0.3)	9 (0.2)		

ckd

outc	Baseline	ICD	le	before matching	after matching

ome	variables	-10	vel	Single drug[a]	Strengthen	p	SMD	Single drug[a]	Strengthen	p	SMD
CKD	gender		0	7786 (50.0)	2249 (52.9)	0.001	0.057	2113 (53.0)	2121 (53.2)	0.875	0.004
			1	7778 (50.0)	2006 (47.1)			1876 (47.0)	1868 (46.8)		
	Paroxysmal and episodic disorders	G40-G47	0	13865 (89.1)	3857 (90.6)	0.004	0.052	3636 (91.2)	3618 (90.7)	0.508	0.016
			1	1699 (10.9)	398 (9.4)			353 (8.8)	371 (9.3)		
	Nerve: Nerve root and plexus disorders	G50-G59	0	15312 (98.4)	4205 (98.8)	0.043	0.038	3942 (98.8)	3944 (98.9)	0.916	0.005
			1	252 (1.6)	50 (1.2)			47 (1.2)	45 (1.1)		
	Conjunctival diseases	H10-H13	0	15298 (98.3)	4170 (98.0)	0.23	0.021	3927 (98.4)	3917 (98.2)	0.433	0.02
			1	266 (1.7)	85 (2.0)			62 (1.6)	72 (1.8)		
	Lens diseases	H25-H28	0	14501 (93.2)	3924 (92.2)	0.035	0.036	3680 (92.3)	3698 (92.7)	0.47	0.017
			1	1063 (6.8)	331 (7.8)			309 (7.7)	291 (7.3)		
	septicemia	A40-A41	0	15546 (99	4240 (99.6)	0.002	0.049	3980 (99	3980 (99.8)	1	<0.001

				.9)				.8)			
			1	18 (0.1)	15 (0.4)			9 (0.2)	9 (0.2)		
	Other diseases of the eye and its adnexa	H55-H59	0	15420 (99.1)	4211 (99.0)	0.575	0.011	3949 (99.0)	3953 (99.1)	0.73	0.01
			1	144 (0.9)	44 (1.0)			40 (1.0)	36 (0.9)		
	Sensory disorders (ear)	H60-H95	0	15132 (97.2)	4141 (97.3)	0.774	0.006	3887 (97.4)	3890 (97.5)	0.886	0.005
			1	432 (2.8)	114 (2.7)			102 (2.6)	99 (2.5)		
	Cardiovascular disease	I00-I15, I20-I28, I30-I51, I60-I79	0	3804 (24.4)	1275 (30.0)	<0.001	0.124	1171 (29.4)	1203 (30.2)	0.448	0.018
			1	11760 (75.6)	2980 (70.0)			2818 (70.6)	2786 (69.8)		
	Hypertension	I10-I15	0	6337 (40.7)	1994 (46.9)	<0.001	0.124	1855 (46.5)	1877 (47.1)	0.637	0.011
			1	9227 (59.3)	2261 (53.1)			2134 (53.5)	2112 (52.9)		
	Acute myocardial infarction	I21-I22	0	15183 (97.6)	4086 (96.0)	<0.001	0.087	3844 (96.4)	3838 (96.2)	0.767	0.008

			1	381 (2.4)	169 (4.0)			145 (3.6)	151 (3.8)		
	Cor pulmonale	I26 - I27	0	15441 (99.2)	4188 (98.4)	<0.001	0.073	3943 (98.8)	3938 (98.7)	0.683	0.011
			1	123 (0.8)	67 (1.6)			46 (1.2)	51 (1.3)		
	Cor pulmonale and pulmonary circulation diseases, including pulmonary embolism, pulmonary hypertension and cor pulmonale	I26 - I28	0	15441 (99.2)	4188 (98.4)	<0.001	0.073	3943 (98.8)	3938 (98.7)	0.683	0.011
			1	123 (0.8)	67 (1.6)			46 (1.2)	51 (1.3)		
	Cardiomyopathy	I42 - I43	0	15534 (99.8)	4237 (99.6)	0.011	0.042	3977 (99.7)	3974 (99.6)	0.7	0.013
			1	30 (0.2)	18 (0.4)			12 (0.3)	15 (0.4)		
	Ischemic stroke	I63 - I64	0	12342 (79.3)	3454 (81.2)	0.007	0.047	3274 (82.1)	3236 (81.1)	0.285	0.025
			1	3222 (20.7)	801 (18.8)			715 (17.9)	753 (18.9)		
	Varicose veins, hemorrhoids	I83 - I86	0	15239 (97.9)	4165 (97.9)	0.961	0.002	3911 (98.0)	3912 (98.1)	1	0.002
			1	325	90			78	77		

				(2.1)	(2.1)			(2.0)	(1.9)		
	Acute upper respiratory tract infection	J00-J06	0	14568 (93.6)	3967 (93.2)	0.406	0.015	3735 (93.6)	3727 (93.4)	0.75	0.008
			1	996 (6.4)	288 (6.8)			254 (6.4)	262 (6.6)		
	asthma	J45-J46	0	15317 (98.4)	4187 (98.4)	1	0.001	3935 (98.6)	3932 (98.6)	0.848	0.006
			1	247 (1.6)	68 (1.6)			54 (1.4)	57 (1.4)		
	respiratory failure	J80, J96	0	15514 (99.7)	4216 (99.1)	<0.001	0.076	3966 (99.4)	3966 (99.4)	1	<0.001
			1	50 (0.3)	39 (0.9)			23 (0.6)	23 (0.6)		
	Other respiratory diseases primarily affecting the interstitial tissue	J80-J84	0	15447 (99.2)	4190 (98.5)	<0.001	0.073	3945 (98.9)	3940 (98.8)	0.677	0.012
			1	117 (0.8)	65 (1.5)			44 (1.1)	49 (1.2)		
	Other pleural diseases	J90-J94	0	15282 (98.2)	4137 (97.2)	<0.001	0.064	3895 (97.6)	3897 (97.7)	0.941	0.003
			1	282 (1.8)	118 (2.8)			94 (2.4)	92 (2.3)		
	Digestive system diseases	K00-K93	0	10517 (67.6)	2645 (62.2)	<0.001	0.114	2562 (64.2)	2554 (64.0)	0.87	0.004
			1	5047	1610 (37.8)			1427	1435 (36.0)		

				(32.4)				(35.8)			
	Cirrhosis	K70, K74	0	15479 (99.5)	4196 (98.6)	<0.001	0.086	3953 (99.1)	3945 (98.9)	0.432	0.02
			1	85 (0.5)	59 (1.4)			36 (0.9)	44 (1.1)		
	Pancreatic diseases	K85-K87	0	15502 (99.6)	4203 (98.8)	<0.001	0.092	3963 (99.3)	3956 (99.2)	0.433	0.02
			1	62 (0.4)	52 (1.2)			26 (0.7)	33 (0.8)		
	Other diseases of the digestive system	K90-K93	0	15397 (98.9)	4145 (97.4)	<0.001	0.113	3921 (98.3)	3923 (98.3)	0.931	0.004
			1	167 (1.1)	110 (2.6)			68 (1.7)	66 (1.7)		
	hepatitis	B15-B19, K73	0	15402 (99.0)	4165 (97.9)	<0.001	0.086	3932 (98.6)	3918 (98.2)	0.247	0.028
			1	162 (1.0)	90 (2.1)			57 (1.4)	71 (1.8)		
	Urticaria and erythema	L50-L54	0	15502 (99.6)	4235 (99.5)	0.61	0.011	3979 (99.7)	3975 (99.6)	0.54	0.018
			1	62 (0.4)	20 (0.5)			10 (0.3)	14 (0.4)		
	arthritis	M00-M25	0	14611 (93.9)	4005 (94.1)	0.573	0.01	3752 (94.1)	3758 (94.2)	0.812	0.006
			1	953 (6.1)	250 (5.9)			237 (5.9)	231 (5.8)		
	Inflammator	M0	0	151	4138	0.37	0.01	388	3888	0.8	0.00

	y polyarthritis	5-M14		76 (97.5)	(97.3)	5	6	4 (97.4)	(97.5)	32	6
			1	388 (2.5)	117 (2.7)			105 (2.6)	101 (2.5)		
	Other back pain	M50-M54	0	14308 (91.9)	3956 (93.0)	0.027	0.039	3708 (93.0)	3712 (93.1)	0.895	0.004
			1	1256 (8.1)	299 (7.0)			281 (7.0)	277 (6.9)		
	Synovial and tendon disorders	M65-M68	0	15503 (99.6)	4243 (99.7)	0.365	0.019	3975 (99.6)	3982 (99.8)	0.19	0.034
			1	61 (0.4)	12 (0.3)			14 (0.4)	7 (0.2)		
	Other soft tissue diseases	M70-M79	0	15280 (98.2)	4179 (98.2)	0.919	0.003	3914 (98.1)	3924 (98.4)	0.443	0.019
			1	284 (1.8)	76 (1.8)			75 (1.9)	65 (1.6)		
	Disorders of bone density and structure	M80-M85	0	15196 (97.6)	4099 (96.3)	<0.001	0.076	3879 (97.2)	3866 (96.9)	0.425	0.019
			1	368 (2.4)	156 (3.7)			110 (2.8)	123 (3.1)		
	Fungal diseases	B35-B49	0	15476 (99.4)	4231 (99.4)	1	<0.001	3968 (99.5)	3969 (99.5)	1	0.004
			1	88 (0.6)	24 (0.6)			21 (0.5)	20 (0.5)		
	Nephritis	N00-N19	0	15162 (97.4)	4076 (95.8)	<0.001	0.09	3857 (96.7)	3848 (96.5)	0.622	0.012

			1	402 (2.6)	179 (4.2)			132 (3.3)	141 (3.5)		
	Urinary system diseases	N30-N39	0	14891 (95.7)	4004 (94.1)	<0.001	0.072	3782 (94.8)	3777 (94.7)	0.841	0.006
			1	673 (4.3)	251 (5.9)			207 (5.2)	212 (5.3)		
	Female genital conditions such as menstruation and menopause	N91-N95	0	15447 (99.2)	4211 (99.0)	0.085	0.03	3960 (99.3)	3955 (99.1)	0.613	0.014
			1	117 (0.8)	44 (1.0)			29 (0.7)	34 (0.9)		
	Pregnancy, childbirth and postpartum period	O00-O99	0	15457 (99.3)	4196 (98.6)	<0.001	0.069	3941 (98.8)	3942 (98.8)	1	0.002
			1	107 (0.7)	59 (1.4)			48 (1.2)	47 (1.2)		
	Other maternal conditions primarily related to pregnancy	O20-O29	0	15511 (99.7)	4218 (99.1)	<0.001	0.068	3961 (99.3)	3962 (99.3)	1	0.003
			1	53 (0.3)	37 (0.9)			28 (0.7)	27 (0.7)		
	Congenital malformations of the urinary system	Q60-Q64	0	15542 (99.9)	4244 (99.7)	0.147	0.026	3982 (99.8)	3979 (99.7)	0.627	0.016
			1	22 (0.1)	11 (0.3)			7 (0.2)	10 (0.3)		
	Symptoms	R00	0	146	4005	1	<0.	377	3766	0.5	0.01

	and signs involving the circulatory and respiratory systems	- R09		51 (94.1)	(94.1)		001	9 (94.7)	(94.4)	53	4
			1	913 (5.9)	250 (5.9)			210 (5.3)	223 (5.6)		
	Symptoms and signs involving cognition, perception, emotional state, and behavior	R40 - R46	0	14814 (95.2)	4072 (95.7)	0.17	0.025	3817 (95.7)	3822 (95.8)	0.824	0.006
			1	750 (4.8)	183 (4.3)			172 (4.3)	167 (4.2)		
	Abnormal blood test, no diagnosis	R70 - R79	0	15234 (97.9)	4100 (96.4)	<0.001	0.091	3855 (96.6)	3865 (96.9)	0.569	0.014
			1	330 (2.1)	155 (3.6)			134 (3.4)	124 (3.1)		
	Abnormal findings on diagnostic imaging and functional studies, no diagnosis	R90 - R94	0	14799 (95.1)	3974 (93.4)	<0.001	0.073	3761 (94.3)	3753 (94.1)	0.738	0.009
			1	765 (4.9)	281 (6.6)			228 (5.7)	236 (5.9)		
	Hip and thigh injuries	S70 - S79	0	15492 (99.5)	4226 (99.3)	0.098	0.029	3966 (99.4)	3964 (99.4)	0.885	0.006
			1	72 (0.5)	29 (0.7)			23 (0.6)	25 (0.6)		
	Digestive system	C15 -	0	15355	4088 (96.1)	<0.001	0.162	3903	3900 (97.8)	0.878	0.005

	cancer	C26		(98.7)				(97.8)			
			1	209 (1.3)	167 (3.9)			86 (2.2)	89 (2.2)		
	Tumor	C00-C99, D00-D48	0	14065 (90.4)	3601 (84.6)	<0.001	0.174	3503 (87.8)	3474 (87.1)	0.344	0.022
			1	1499 (9.6)	654 (15.4)			486 (12.2)	515 (12.9)		
	Malignant tumors	C00-C98	0	14778 (94.9)	3823 (89.8)	<0.001	0.193	3702 (92.8)	3685 (92.4)	0.494	0.016
			1	786 (5.1)	432 (10.2)			287 (7.2)	304 (7.6)		
	Other malignant tumors	C00-C14, C17, C26-C31, C37-C39, C43-C49, C51-C52,	0	15155 (97.4)	4016 (94.4)	<0.001	0.151	3841 (96.3)	3833 (96.1)	0.682	0.01

		C57-C60, C62-C63, C68-C69, C73-C76, C80, C97									
			1	409 (2.6)	239 (5.6)			148 (3.7)	156 (3.9)		
	Urinary tract malignancy?	C64-C68	0	15506 (99.6)	4237 (99.6)	0.74	0.008	3971 (99.5)	3973 (99.6)	0.864	0.008
			1	58 (0.4)	18 (0.4)			18 (0.5)	16 (0.4)		
	Malignant tumors of endocrine glands	C73-C75	0	15496 (99.6)	4234 (99.5)	0.719	0.008	3975 (99.6)	3971 (99.5)	0.595	0.016
			1	68 (0.4)	21 (0.5)			14 (0.4)	18 (0.5)		
	Aplastic anemia	D60-D61	0	15531 (99.8)	4243 (99.7)	0.504	0.014	3979 (99.7)	3980 (99.8)	1	0.005
			1	33 (0.2)	12 (0.3)			10 (0.3)	9 (0.2)		
	Uterine malignancy	C53-C55	0	15522 (99	4227 (99.3)	<0.001	0.057	3971 (99	3969 (99.5)	0.871	0.007

				.7)				.5)			
			1	42 (0.3)	28 (0.7)			18 (0.5)	20 (0.5)		
	Other types of heart disease	I30-I52	0	11988 (77.0)	3245 (76.3)	0.307	0.018	3075 (77.1)	3076 (77.1)	1	0.001
			1	3576 (23.0)	1010 (23.7)			914 (22.9)	913 (22.9)		
	Lung diseases due to foreign substances	J60-J70	0	15537 (99.8)	4237 (99.6)	0.004	0.046	3977 (99.7)	3977 (99.7)	1	<0.001
			1	27 (0.2)	18 (0.4)			12 (0.3)	12 (0.3)		
	Other diseases of the respiratory system	J95-J99	0	14963 (96.1)	4010 (94.2)	<0.001	0.089	3801 (95.3)	3797 (95.2)	0.875	0.005
			1	601 (3.9)	245 (5.8)			188 (4.7)	192 (4.8)		
	Nutritional anemia	D50-D53	0	15510 (99.7)	4217 (99.1)	<0.001	0.07	3965 (99.4)	3962 (99.3)	0.779	0.009
			1	54 (0.3)	38 (0.9)			24 (0.6)	27 (0.7)		
	anemia	D50-D64	0	15198 (97.6)	4046 (95.1)	<0.001	0.137	3860 (96.8)	3842 (96.3)	0.298	0.025
			1	366 (2.4)	209 (4.9)			129 (3.2)	147 (3.7)		
	Other arthritis	M12-M14	0	15366 (98.7)	4190 (98.5)	0.224	0.022	3935 (98.6)	3937 (98.7)	0.922	0.004
			1	198	65			54	52		

				(1.3)	(1.5)			(1.4)	(1.3)		
	Diseases of the blood and blood-forming organs and certain diseases involving the immune system	D50-D89	0	14796 (95.1)	3913 (92.0)	<0.001	0.126	3744 (93.9)	3727 (93.4)	0.463	0.017
			1	768 (4.9)	342 (8.0)			245 (6.1)	262 (6.6)		
	Bone disease and osteomalacia	M80-M94	0	15075 (96.9)	4061 (95.4)	<0.001	0.074	3843 (96.3)	3831 (96.0)	0.52	0.016
			1	489 (3.1)	194 (4.6)			146 (3.7)	158 (4.0)		
	Kidney and bladder diseases	N23-N29, N31-N33, N35-N39, N41-N64	0	13985 (89.9)	3713 (87.3)	<0.001	0.082	3526 (88.4)	3514 (88.1)	0.702	0.009
			1	1579 (10.1)	542 (12.7)			463 (11.6)	475 (11.9)		
	Nutritional disorders other than lipidemia	E40-E77,	0	14696 (94.4)	3906 (91.8)	<0.001	0.104	3730 (93.5)	3718 (93.2)	0.621	0.012

		E79 - E90									
			1	868 (5.6)	349 (8.2)			259 (6.5)	271 (6.8)		
	Other nutritional deficiencies	E50 - E64	0	15531 (99.8)	4242 (99.7)	0.345	0.018	3979 (99.7)	3978 (99.7)	1	0.005
			1	33 (0.2)	13 (0.3)			10 (0.3)	11 (0.3)		
	Obesity and other overnutrition	E65 - E68	0	15498 (99.6)	4207 (98.9)	<0.001	0.08	3961 (99.3)	3961 (99.3)	1	<0.001
			1	66 (0.4)	48 (1.1)			28 (0.7)	28 (0.7)		
	Metabolic disorders	E70 - E90	0	12995 (83.5)	3405 (80.0)	<0.001	0.09	3279 (82.2)	3238 (81.2)	0.247	0.027
			1	2569 (16.5)	850 (20.0)			710 (17.8)	751 (18.8)		
	Neurotic , stress-related and somatoform disorders	F40 - F48	0	14695 (94.4)	3928 (92.3)	<0.001	0.085	3721 (93.3)	3701 (92.8)	0.403	0.02
			1	869 (5.6)	327 (7.7)			268 (6.7)	288 (7.2)		
	Penicillin V Potassium		0	15500 (99.6)	4207 (98.9)	<0.001	0.082	3952 (99.1)	3943 (98.8)	0.377	0.022
			1	64 (0.4)	48 (1.1)			37 (0.9)	46 (1.2)		
	Cefixime		0	15520 (99	4254 (100.0)	0.003	0.066	3987 (99	3988 (100.0)	1	0.013

				.7)				.9)			
			1	44 (0.3)	1 (0.0)			2 (0.1)	1 (0.0)		
	Cefuroxime		0	15467 (99.4)	4243 (99.7)	0.011	0.051	3981 (99.8)	3977 (99.7)	0.502	0.02
			1	97 (0.6)	12 (0.3)			8 (0.2)	12 (0.3)		
	Vitamin B1		0	15382 (98.8)	4238 (99.6)	<0.001	0.087	3976 (99.7)	3975 (99.6)	1	0.004
			1	182 (1.2)	17 (0.4)			13 (0.3)	14 (0.4)		
	Vitamin C		0	15288 (98.2)	4140 (97.3)	<0.001	0.063	3912 (98.1)	3897 (97.7)	0.276	0.026
			1	276 (1.8)	115 (2.7)			77 (1.9)	92 (2.3)		
	Compound electrolyte		0	15528 (99.8)	4237 (99.6)	0.05	0.034	3978 (99.7)	3975 (99.6)	0.689	0.013
			1	36 (0.2)	18 (0.4)			11 (0.3)	14 (0.4)		
	Potassium chloride		0	15097 (97.0)	4055 (95.3)	<0.001	0.088	3849 (96.5)	3843 (96.3)	0.763	0.008
			1	467 (3.0)	200 (4.7)			140 (3.5)	146 (3.7)		
	Sodium chloride		0	9617 (61.8)	2660 (62.5)	0.398	0.015	2609 (65.4)	2561 (64.2)	0.271	0.025
			1	5947 (38	1595 (37.5)			1380 (34	1428 (35.8)		

				.2)				.6)			
	Invert Sugar Electrolytes		0	15546 (99.9)	4244 (99.7)	0.053	0.033	3987 (99.9)	3985 (99.9)	0.683	0.018
			1	18 (0.1)	11 (0.3)			2 (0.1)	4 (0.1)		
	Coenzyme Q10		0	15506 (99.6)	4249 (99.9)	0.027	0.046	3984 (99.9)	3983 (99.8)	1	0.007
			1	58 (0.4)	6 (0.1)			5 (0.1)	6 (0.2)		
	Levothyroxine		0	15469 (99.4)	4243 (99.7)	0.013	0.049	3974 (99.6)	3977 (99.7)	0.7	0.013
			1	95 (0.6)	12 (0.3)			15 (0.4)	12 (0.3)		
	Loratadine		0	15515 (99.7)	4253 (100.0)	0.004	0.063	3988 (100.0)	3987 (99.9)	1	0.013
			1	49 (0.3)	2 (0.0)			1 (0.0)	2 (0.1)		
	Oxaliplatin		0	15535 (99.8)	4251 (99.9)	0.273	0.025	3986 (99.9)	3985 (99.9)	1	0.008
			1	29 (0.2)	4 (0.1)			3 (0.1)	4 (0.1)		
	Edaravone		0	15490 (99.5)	4229 (99.4)	0.325	0.018	3968 (99.5)	3965 (99.4)	0.765	0.01
			1	74 (0.5)	26 (0.6)			21 (0.5)	24 (0.6)		
	Aspirin clears heat		0	14676 (94	4110 (96.6)	<0.001	0.11	3879 (97	3849 (96.5)	0.062	0.043

				.3)				.2)			
			1	888 (5.7)	145 (3.4)			110 (2.8)	140 (3.5)		
	Compound paracetamol		0	15394 (98.9)	4230 (99.4)	0.004	0.055	3968 (99.5)	3965 (99.4)	0.765	0.01
			1	170 (1.1)	25 (0.6)			21 (0.5)	24 (0.6)		
	Isosorbide dinitrate		0	15366 (98.7)	4198 (98.7)	0.788	0.006	3955 (99.1)	3946 (98.9)	0.36	0.023
			1	198 (1.3)	57 (1.3)			34 (0.9)	43 (1.1)		
	Amlodipine		0	14557 (93.5)	4104 (96.5)	<0.001	0.134	3824 (95.9)	3842 (96.3)	0.326	0.023
			1	1007 (6.5)	151 (3.5)			165 (4.1)	147 (3.7)		
	Felodipine		0	15218 (97.8)	4222 (99.2)	<0.001	0.119	3958 (99.2)	3957 (99.2)	1	0.003
			1	346 (2.2)	33 (0.8)			31 (0.8)	32 (0.8)		
	Nifedipine		0	13490 (86.7)	3923 (92.2)	<0.001	0.18	3691 (92.5)	3675 (92.1)	0.528	0.015
			1	2074 (13.3)	332 (7.8)			298 (7.5)	314 (7.9)		
	Fenofibrate		0	15405 (99.0)	4241 (99.7)	<0.001	0.085	3977 (99.7)	3976 (99.7)	1	0.004
			1	159	14			12	13		

				(1.0)	(0.3)			(0.3)	(0.3)		
	Lovastatin		0	15482 (99.5)	4251 (99.9)	<0.001	0.078	3982 (99.8)	3985 (99.9)	0.546	0.02
			1	82 (0.5)	4 (0.1)			7 (0.2)	4 (0.1)		
	aspirin		0	11106 (71.4)	3570 (83.9)	<0.001	0.304	3319 (83.2)	3336 (83.6)	0.63	0.011
			1	4458 (28.6)	685 (16.1)			670 (16.8)	653 (16.4)		
	Pantoprazole		0	15169 (97.5)	4072 (95.7)	<0.001	0.097	3873 (97.1)	3862 (96.8)	0.515	0.016
			1	395 (2.5)	183 (4.3)			116 (2.9)	127 (3.2)		
	Metoclopramide		0	15471 (99.4)	4227 (99.3)	0.735	0.008	3973 (99.6)	3969 (99.5)	0.616	0.015
			1	93 (0.6)	28 (0.7)			16 (0.4)	20 (0.5)		
	Torsemide		0	15488 (99.5)	4216 (99.1)	0.002	0.051	3967 (99.4)	3968 (99.5)	1	0.003
			1	76 (0.5)	39 (0.9)			22 (0.6)	21 (0.5)		
	Furosemide		0	15190 (97.6)	4129 (97.0)	0.045	0.035	3893 (97.6)	3900 (97.8)	0.655	0.012
			1	374 (2.4)	126 (3.0)			96 (2.4)	89 (2.2)		
	Tamsulosin		0	154	4222	0.23	0.02	396	3967	0.8	0.00

				71 (99.4)	(99.2)	6	2	5 (99.4)	(99.4)	82	7
			1	93 (0.6)	33 (0.8)			24 (0.6)	22 (0.6)		
	Hemocoagulase		0	15543 (99.9)	4239 (99.6)	0.002	0.048	3982 (99.8)	3983 (99.8)	1	0.006
			1	21 (0.1)	16 (0.4)			7 (0.2)	6 (0.2)		
	Low molecular weight heparin calcium		0	15313 (98.4)	4160 (97.8)	0.008	0.045	3921 (98.3)	3915 (98.1)	0.672	0.011
			1	251 (1.6)	95 (2.2)			68 (1.7)	74 (1.9)		
	Cold and heat		0	15480 (99.5)	4248 (99.8)	0.002	0.063	3984 (99.9)	3983 (99.8)	1	0.007
			1	84 (0.5)	7 (0.2)			5 (0.1)	6 (0.2)		
	Cold Remedy		0	15513 (99.7)	4252 (99.9)	0.007	0.058	3985 (99.9)	3986 (99.9)	1	0.008
			1	51 (0.3)	3 (0.1)			4 (0.1)	3 (0.1)		
	Anti-inflammatory and choleretic		0	15539 (99.8)	4254 (100.0)	0.051	0.045	3987 (99.9)	3988 (100.0)	1	0.013
			1	25 (0.2)	1 (0.0)			2 (0.1)	1 (0.0)		
	Xinhuang		0	15496 (99.6)	4247 (99.8)	0.029	0.045	3983 (99.8)	3983 (99.8)	1	<0.001
			1	68	8			6	6		

				(0.4)	(0.2)			(0.2)	(0.2)		
	Sanguisorba officinalis		0	15548 (99.9)	4242 (99.7)	0.005	0.045	3982 (99.8)	3982 (99.8)	1	<0.001
			1	16 (0.1)	13 (0.3)			7 (0.2)	7 (0.2)		
	Ginkgo biloba Chinese medicine		0	14957 (96.1)	4167 (97.9)	<0.001	0.108	3896 (97.7)	3905 (97.9)	0.543	0.015
			1	607 (3.9)	88 (2.1)			93 (2.3)	84 (2.1)		
	Salvianolate		0	15518 (99.7)	4234 (99.5)	0.068	0.032	3970 (99.5)	3971 (99.5)	1	0.004
			1	46 (0.3)	21 (0.5)			19 (0.5)	18 (0.5)		
	Compound Danshen		0	14588 (93.7)	4119 (96.8)	<0.001	0.145	3864 (96.9)	3857 (96.7)	0.704	0.01
			1	976 (6.3)	136 (3.2)			125 (3.1)	132 (3.3)		
	Safflower yellow		0	15478 (99.4)	4229 (99.4)	0.737	0.008	3968 (99.5)	3967 (99.4)	1	0.003
			1	86 (0.6)	26 (0.6)			21 (0.5)	22 (0.6)		
	Activate blood circulation and relieve pain		0	15517 (99.7)	4252 (99.9)	0.013	0.054	3988 (100.0)	3986 (99.9)	0.617	0.022
			1	47 (0.3)	3 (0.1)			1 (0.0)	3 (0.1)		
	Salvia miltiorrhiza		0	15256 (99.0)	4211 (99.0)	<0.001	0.078	3942	3947 (98.9)	0.67	0.012

				(98.0)				(98.8)			
			1	308 (2.0)	44 (1.0)			47 (1.2)	42 (1.1)		
	Intraocular lens implantation		0	15529 (99.8)	4235 (99.5)	0.011	0.042	3973 (99.6)	3976 (99.7)	0.71	0.012
			1	35 (0.2)	20 (0.5)			16 (0.4)	13 (0.3)		
	Vitreous surgery		0	15547 (99.9)	4246 (99.8)	0.163	0.026	3987 (99.9)	3989 (100.0)	0.479	0.032
			1	17 (0.1)	9 (0.2)			2 (0.1)	0 (0.0)		
	Diagnostic procedures of the stomach		0	15526 (99.8)	4227 (99.3)	<0.001	0.062	3971 (99.5)	3965 (99.4)	0.439	0.021
			1	38 (0.2)	28 (0.7)			18 (0.5)	24 (0.6)		
	Soft tissue x-rays of the face, head, and neck		0	15504 (99.6)	4220 (99.2)	<0.001	0.056	3958 (99.2)	3962 (99.3)	0.693	0.012
			1	60 (0.4)	35 (0.8)			31 (0.8)	27 (0.7)		
	Abdominal soft tissue X-ray examination		0	15536 (99.8)	4233 (99.5)	<0.001	0.057	3978 (99.7)	3980 (99.8)	0.823	0.01
			1	28 (0.2)	22 (0.5)			11 (0.3)	9 (0.2)		

Cancer

outcome	Baseline variables	ICD-10	level	before matching				after matching			
				Single	Strengthen	p	SMD	Single	Strengthen	p	SMD

				drug[a]				drug[a]			
cancer	gender		0	7719 (50.0)	2184 (53.2)	<0.001	0.065	2047 (52.7)	2068 (53.3)	0.649	0.011
			1	7722 (50.0)	1920 (46.8)			1836 (47.3)	1815 (46.7)		
	Paroxysmal and episodic disorders	G40 - G47	0	13753 (89.1)	3719 (90.6)	0.005	0.051	3544 (91.3)	3517 (90.6)	0.304	0.024
			1	1688 (10.9)	385 (9.4)			339 (8.7)	366 (9.4)		
	Nerve: Nerve root and plexus disorders	G50 - G59	0	15190 (98.4)	4057 (98.9)	0.031	0.041	3835 (98.8)	3837 (98.8)	0.917	0.005
			1	251 (1.6)	47 (1.1)			48 (1.2)	46 (1.2)		
	Sensory diseases (eye)	H00 - H59	0	13644 (88.4)	3574 (87.1)	0.027	0.039	3403 (87.6)	3404 (87.7)	1	0.001
			1	1797 (11.6)	530 (12.9)			480 (12.4)	479 (12.3)		
	Conjunctival diseases	H10 - H13	0	15182 (98.3)	4023 (98.0)	0.221	0.022	3822 (98.4)	3812 (98.2)	0.429	0.02
			1	259 (1.7)	81 (2.0)			61 (1.6)	71 (1.8)		
	Lens diseases	H25 - H28	0	14390 (93.2)	3783 (92.2)	0.026	0.039	3584 (92.3)	3600 (92.7)	0.518	0.016
			1	105	321			299	283		

				1 (6.8)	(7.8)			(7.7)	(7.3)		
	septicemia	A40 - A41	0	15423 (99.9)	4089 (99.6)	0.001	0.051	3874 (99.8)	3874 (99.8)	1	<0.001
			1	18 (0.1)	15 (0.4)			9 (0.2)	9 (0.2)		
	Eye muscle disorders, binocular movements, accommodation and refraction	H49 - H52	0	15254 (98.8)	4058 (98.9)	0.695	0.008	3835 (98.8)	3841 (98.9)	0.596	0.014
			1	187 (1.2)	46 (1.1)			48 (1.2)	42 (1.1)		
	Other diseases of the eye and its adnexa	H55 - H59	0	15298 (99.1)	4062 (99.0)	0.63	0.01	3848 (99.1)	3845 (99.0)	0.814	0.008
			1	143 (0.9)	42 (1.0)			35 (0.9)	38 (1.0)		
	Sensory disorders (ear)	H60 - H95	0	15010 (97.2)	3994 (97.3)	0.74	0.007	3794 (97.7)	3783 (97.4)	0.461	0.018
			1	431 (2.8)	110 (2.7)			89 (2.3)	100 (2.6)		
	Hearing loss that begins in adulthood	H90 - H91	0	15300 (99.1)	4073 (99.2)	0.385	0.017	3858 (99.4)	3854 (99.3)	0.682	0.012
			1	141 (0.9)	31 (0.8)			25 (0.6)	29 (0.7)		
	Cardiovascular disease	I00 - I15, I20 -	0	3774 (24.4)	1225 (29.8)	<0.001	0.122	1166 (30.0)	1171 (30.2)	0.921	0.003

		I28, I30 - I51, I60 - I79									
			1	11667 (75.6)	2879 (70.2)			2717 (70.0)	2712 (69.8)		
	Hypertension	I10 - I15	0	6291 (40.7)	1925 (46.9)	<0.001	0.124	1823 (46.9)	1835 (47.3)	0.803	0.006
			1	9150 (59.3)	2179 (53.1)			2060 (53.1)	2048 (52.7)		
	Acute myocardial infarction	I21 - I22	0	15060 (97.5)	3932 (95.8)	<0.001	0.096	3733 (96.1)	3735 (96.2)	0.953	0.003
			1	381 (2.5)	172 (4.2)			150 (3.9)	148 (3.8)		
	Cor pulmonale	I26 - I27	0	15321 (99.2)	4036 (98.3)	<0.001	0.08	3840 (98.9)	3830 (98.6)	0.355	0.023
			1	120 (0.8)	68 (1.7)			43 (1.1)	53 (1.4)		
	Cor pulmonale and pulmonary circulation diseases, including pulmonary embolism, pulmonary hypertension and cor pulmonale	I26 - I28	0	15321 (99.2)	4036 (98.3)	<0.001	0.08	3840 (98.9)	3830 (98.6)	0.355	0.023

			1	120 (0.8)	68 (1.7)			43 (1.1)	53 (1.4)		
	Cardiomyopathy	I42-I43	0	15410 (99.8)	4084 (99.5)	0.002	0.049	3870 (99.7)	3867 (99.6)	0.71	0.013
			1	31 (0.2)	20 (0.5)			13 (0.3)	16 (0.4)		
	Ischemic stroke	I63-I64	0	12233 (79.2)	3328 (81.1)	0.009	0.047	3186 (82.0)	3149 (81.1)	0.292	0.025
			1	3208 (20.8)	776 (18.9)			697 (18.0)	734 (18.9)		
	Aneurysm (aortic and other)	I71-I72	0	15415 (99.8)	4091 (99.7)	0.09	0.03	3873 (99.7)	3873 (99.7)	1	<0.001
			1	26 (0.2)	13 (0.3)			10 (0.3)	10 (0.3)		
	Varicose veins, hemorrhoids	I83-I86	0	15121 (97.9)	4017 (97.9)	0.898	0.003	3807 (98.0)	3810 (98.1)	0.869	0.006
			1	320 (2.1)	87 (2.1)			76 (2.0)	73 (1.9)		
	Acute upper respiratory tract infection	J00-J06	0	14456 (93.6)	3828 (93.3)	0.444	0.014	3644 (93.8)	3626 (93.4)	0.43	0.019
			1	985 (6.4)	276 (6.7)			239 (6.2)	257 (6.6)		
	asthma	J45-J46	0	15197 (98.4)	4041 (98.5)	0.892	0.004	3836 (98.8)	3827 (98.6)	0.427	0.02
			1	244 (1.6)	63 (1.5)			47 (1.2)	56 (1.4)		

	respiratory failure	J80, J96	0	15389 (99.7)	4069 (99.1)	<0.001	0.067	3861 (99.4)	3854 (99.3)	0.399	0.022
			1	52 (0.3)	35 (0.9)			22 (0.6)	29 (0.7)		
	Other respiratory diseases primarily affecting the interstitial tissue	J80 - J84	0	15327 (99.3)	4041 (98.5)	<0.001	0.075	3837 (98.8)	3832 (98.7)	0.683	0.012
			1	114 (0.7)	63 (1.5)			46 (1.2)	51 (1.3)		
	Other pleural diseases	J90 - J94	0	15158 (98.2)	3989 (97.2)	<0.001	0.064	3799 (97.8)	3788 (97.6)	0.45	0.019
			1	283 (1.8)	115 (2.8)			84 (2.2)	95 (2.4)		
	Digestive system diseases	K00 - K93	0	10451 (67.7)	2584 (63.0)	<0.001	0.099	2528 (65.1)	2492 (64.2)	0.406	0.019
			1	4990 (32.3)	1520 (37.0)			1355 (34.9)	1391 (35.8)		
	Peptic ulcer	K25 - K28	0	15225 (98.6)	4052 (98.7)	0.569	0.011	3837 (98.8)	3833 (98.7)	0.758	0.009
			1	216 (1.4)	52 (1.3)			46 (1.2)	50 (1.3)		
	Gastric/duodenal ulcer	K25 - K28, K51	0	15198 (98.4)	4041 (98.5)	0.915	0.003	3828 (98.6)	3825 (98.5)	0.85	0.006
			1	243 (1.	63 (1.5)			55 (1.	58 (1.5)		

				6)				4)			
	Pancreatic diseases	K85-K87	0	15380 (99.6)	4059 (98.9)	<0.001	0.082	3856 (99.3)	3856 (99.3)	1	<0.001
			1	61 (0.4)	45 (1.1)			27 (0.7)	27 (0.7)		
	Other diseases of the digestive system	K90-K93	0	15277 (98.9)	4002 (97.5)	<0.001	0.108	3816 (98.3)	3814 (98.2)	0.931	0.004
			1	164 (1.1)	102 (2.5)			67 (1.7)	69 (1.8)		
	hepatitis	B15-B19, K73	0	15283 (99.0)	4022 (98.0)	<0.001	0.08	3834 (98.7)	3820 (98.4)	0.216	0.03
			1	158 (1.0)	82 (2.0)			49 (1.3)	63 (1.6)		
	Urticaria and erythema	L50-L54	0	15381 (99.6)	4085 (99.5)	0.597	0.011	3873 (99.7)	3867 (99.6)	0.326	0.027
			1	60 (0.4)	19 (0.5)			10 (0.3)	16 (0.4)		
	Inflammatory polyarthritis	M05-M14	0	15065 (97.6)	3993 (97.3)	0.353	0.017	3792 (97.7)	3782 (97.4)	0.511	0.017
			1	376 (2.4)	111 (2.7)			91 (2.3)	101 (2.6)		
	Other back pain	M50-M54	0	14206 (92.0)	3817 (93.0)	0.035	0.038	3631 (93.5)	3615 (93.1)	0.496	0.016
			1	1235 (8.0)	287 (7.0)			252 (6.5)	268 (6.9)		
	Muscle	M6	0	154	4092	0.03	0.03	387	3874	1	0.00

	disorders	0-M63		21 (99.9)	(99.7)	8	6	3 (99.7)	(99.8)		5
			1	20 (0.1)	12 (0.3)			10 (0.3)	9 (0.2)		
	Synovial and tendon disorders	M65-M68	0	15381 (99.6)	4095 (99.8)	0.14	0.031	3873 (99.7)	3875 (99.8)	0.813	0.011
			1	60 (0.4)	9 (0.2)			10 (0.3)	8 (0.2)		
	Other soft tissue diseases	M70-M79	0	15155 (98.1)	4031 (98.2)	0.806	0.006	3809 (98.1)	3817 (98.3)	0.55	0.015
			1	286 (1.9)	73 (1.8)			74 (1.9)	66 (1.7)		
	Disorders of bone density and structure	M80-M85	0	15079 (97.7)	3954 (96.3)	<0.001	0.077	3773 (97.2)	3766 (97.0)	0.686	0.011
			1	362 (2.3)	150 (3.7)			110 (2.8)	117 (3.0)		
	Nephritis	N00-N01, N03-N05, N08, N10-N12, N15-N16, N1	0	15169 (98.2)	3962 (96.5)	<0.001	0.107	3781 (97.4)	3766 (97.0)	0.337	0.023

		8									
			1	272 (1.8)	142 (3.5)			102 (2.6)	117 (3.0)		
	Fungal diseases	B35 - B49	0	15354 (99.4)	4079 (99.4)	0.819	0.006	3865 (99.5)	3861 (99.4)	0.634	0.014
			1	87 (0.6)	25 (0.6)			18 (0.5)	22 (0.6)		
	Nephritis and kidney disease	N00- N19	0	15014 (97.2)	3899 (95.0)	<0.001	0.116	3729 (96.0)	3717 (95.7)	0.53	0.016
			1	427 (2.8)	205 (5.0)			154 (4.0)	166 (4.3)		
	Kidney disease	I12 - I13, N03- N04, N17- N19, R34	0	15106 (97.8)	3932 (95.8)	<0.001	0.115	3760 (96.8)	3749 (96.5)	0.526	0.016
			1	335 (2.2)	172 (4.2)			123 (3.2)	134 (3.5)		
	Chronic kidney disease	N18	0	15319 (99.2)	4032 (98.2)	<0.001	0.086	3836 (98.8)	3828 (98.6)	0.485	0.018
			1	122 (0.8)	72 (1.8)			47 (1.2)	55 (1.4)		
	Urinary system diseases	N30- N39	0	14777 (95.7)	3866 (94.2)	<0.001	0.068	3685 (94.9)	3669 (94.5)	0.448	0.018
			1	664 (4.	238 (5.8)			198 (5.	214 (5.5)		

				3)				1)			
	Pregnancy, childbirth and postpartum period	O00-O99	0	15336 (99.3)	4044 (98.5)	<0.001	0.076	3843 (99.0)	3835 (98.8)	0.453	0.019
			1	105 (0.7)	60 (1.5)			40 (1.0)	48 (1.2)		
	Other maternal conditions primarily related to pregnancy	O20-O29	0	15388 (99.7)	4067 (99.1)	<0.001	0.071	3862 (99.5)	3854 (99.3)	0.321	0.026
			1	53 (0.3)	37 (0.9)			21 (0.5)	29 (0.7)		
	Congenital malformations of the urinary system	Q60-Q64	0	15419 (99.9)	4093 (99.7)	0.127	0.028	3875 (99.8)	3875 (99.8)	1	<0.001
			1	22 (0.1)	11 (0.3)			8 (0.2)	8 (0.2)		
	Symptoms and signs involving the circulatory and respiratory systems	R00-R09	0	14531 (94.1)	3864 (94.2)	0.942	0.002	3701 (95.3)	3663 (94.3)	0.058	0.044
			1	910 (5.9)	240 (5.8)			182 (4.7)	220 (5.7)		
	Symptoms and signs involving cognition, perception, emotional state, and behavior	R40-R46	0	14702 (95.2)	3927 (95.7)	0.218	0.023	3730 (96.1)	3719 (95.8)	0.566	0.014
			1	739 (4.	177 (4.3)			153 (3.	164 (4.2)		

				8)				9)			
	Abnormal blood test, no diagnosis	R70 - R79	0	15112 (97.9)	3957 (96.4)	<0.001	0.087	3766 (97.0)	3762 (96.9)	0.843	0.006
			1	329 (2.1)	147 (3.6)			117 (3.0)	121 (3.1)		
	Abnormal findings on diagnostic imaging and functional studies, no diagnosis	R90 - R94	0	14705 (95.2)	3855 (93.9)	0.001	0.057	3684 (94.9)	3669 (94.5)	0.479	0.017
			1	736 (4.8)	249 (6.1)			199 (5.1)	214 (5.5)		
	Hip and thigh injuries	S70 - S79	0	15367 (99.5)	4072 (99.2)	0.027	0.038	3860 (99.4)	3857 (99.3)	0.774	0.01
			1	74 (0.5)	32 (0.8)			23 (0.6)	26 (0.7)		
	Digestive system cancer	C15 - C26	0	15244 (98.7)	4000 (97.5)	<0.001	0.092	3818 (98.3)	3805 (98.0)	0.311	0.025
			1	197 (1.3)	104 (2.5)			65 (1.7)	78 (2.0)		
	Surgical and medical complications, not otherwise classified	T80 - T88	0	15388 (99.7)	4076 (99.3)	0.004	0.047	3863 (99.5)	3862 (99.5)	1	0.004
			1	53 (0.3)	28 (0.7)			20 (0.5)	21 (0.5)		
	Drug use-induced mental disorders	F10 - F16, F18 -	0	15425 (99.9)	4094 (99.8)	0.052	0.034	3879 (99.9)	3877 (99.8)	0.752	0.014

		F19									
			1	16 (0.1)	10 (0.2)			4 (0.1)	6 (0.2)		
	Carcinoma in situ	D00-D02, D04-D07, D09	0	15387 (99.7)	4074 (99.3)	0.001	0.052	3860 (99.4)	3860 (99.4)	1	<0.001
			1	54 (0.3)	30 (0.7)			23 (0.6)	23 (0.6)		
	Tumor in situ	D00-D09	0	15387 (99.7)	4074 (99.3)	0.001	0.052	3860 (99.4)	3860 (99.4)	1	<0.001
			1	54 (0.3)	30 (0.7)			23 (0.6)	23 (0.6)		
	Other types of heart disease	I30-I52	0	11889 (77.0)	3118 (76.0)	0.175	0.024	2992 (77.1)	2983 (76.8)	0.829	0.006
			1	3552 (23.0)	986 (24.0)			891 (22.9)	900 (23.2)		
	Lung diseases due to foreign substances	J60-J70	0	15415 (99.8)	4087 (99.6)	0.005	0.046	3871 (99.7)	3870 (99.7)	1	0.005
			1	26 (0.2)	17 (0.4)			12 (0.3)	13 (0.3)		
	Other diseases of the respiratory system	J95-J99	0	14843 (96.1)	3866 (94.2)	<0.001	0.09	3693 (95.1)	3681 (94.8)	0.569	0.014
			1	598 (3.9)	238 (5.8)			190 (4.9)	202 (5.2)		

	anemia	D50-D64	0	15079 (97.7)	3910 (95.3)	<0.001	0.129	3757 (96.8)	3736 (96.2)	0.218	0.029
			1	362 (2.3)	194 (4.7)			126 (3.2)	147 (3.8)		
	Other arthritis	M12-M14	0	15251 (98.8)	4042 (98.5)	0.181	0.024	3837 (98.8)	3829 (98.6)	0.481	0.018
			1	190 (1.2)	62 (1.5)			46 (1.2)	54 (1.4)		
	Diseases of the blood and blood-forming organs and certain diseases involving the immune system	D50-D89	0	14681 (95.1)	3786 (92.3)	<0.001	0.116	3644 (93.8)	3626 (93.4)	0.43	0.019
			1	760 (4.9)	318 (7.7)			239 (6.2)	257 (6.6)		
	Bone disease and osteomalacia	M80-M94	0	14957 (96.9)	3918 (95.5)	<0.001	0.073	3737 (96.2)	3733 (96.1)	0.859	0.005
			1	484 (3.1)	186 (4.5)			146 (3.8)	150 (3.9)		
	Kidney and bladder diseases	N23-N29, N31-N33, N35-N39,	0	13885 (89.9)	3588 (87.4)	<0.001	0.079	3434 (88.4)	3417 (88.0)	0.573	0.014

		N41-N64									
			1	1556 (10.1)	516 (12.6)			449 (11.6)	466 (12.0)		
	People who come into contact with health care institutions for examinations and investigations	Z00-Z13	0	15001 (97.2)	3955 (96.4)	0.011	0.044	3788 (97.6)	3757 (96.8)	0.041	0.048
			1	440 (2.8)	149 (3.6)			95 (2.4)	126 (3.2)		
	Other endocrine gland diseases	E20-E35	0	15325 (99.2)	4063 (99.0)	0.138	0.027	3854 (99.3)	3851 (99.2)	0.797	0.009
			1	116 (0.8)	41 (1.0)			29 (0.7)	32 (0.8)		
	Nutritional disorders other than lipidemia	E40-E77, E79-E90	0	14581 (94.4)	3766 (91.8)	<0.001	0.105	3614 (93.1)	3613 (93.0)	1	0.001
			1	860 (5.6)	338 (8.2)			269 (6.9)	270 (7.0)		
	Other nutritional deficiencies	E50-E64	0	15407 (99.8)	4092 (99.7)	0.505	0.014	3875 (99.8)	3872 (99.7)	0.646	0.016
			1	34 (0.2)	12 (0.3)			8 (0.2)	11 (0.3)		
	Obesity and	E65	0	153	4057	<0.	0.08	385	3852	0.6	0.01

	other overnutrition	- E68		74 (99.6)	(98.9)	001		6 (99.3)	(99.2)	93	2
			1	67 (0.4)	47 (1.1)			27 (0.7)	31 (0.8)		
	Metabolic disorders	E70 - E90	0	12891 (83.5)	3276 (79.8)	<0.001	0.095	3153 (81.2)	3143 (80.9)	0.794	0.007
			1	2550 (16.5)	828 (20.2)			730 (18.8)	740 (19.1)		
	Mental and behavioral disorders due to psychoactive substance use	F10 - F19	0	15425 (99.9)	4094 (99.8)	0.052	0.034	3879 (99.9)	3877 (99.8)	0.752	0.014
			1	16 (0.1)	10 (0.2)			4 (0.1)	6 (0.2)		
	Neurotic , stress-related and somatoform disorders	F40 - F48	0	14579 (94.4)	3788 (92.3)	<0.001	0.085	3592 (92.5)	3601 (92.7)	0.728	0.009
			1	862 (5.6)	316 (7.7)			291 (7.5)	282 (7.3)		
	Penicillin V Potassium		0	15377 (99.6)	4061 (99.0)	<0.001	0.074	3842 (98.9)	3845 (99.0)	0.821	0.008
			1	64 (0.4)	43 (1.0)			41 (1.1)	38 (1.0)		
	Cefixime		0	15399 (99.7)	4102 (100.0)	0.013	0.056	3881 (99.9)	3881 (99.9)	1	<0.001
			1	42 (0.3)	2 (0.0)			2 (0.1)	2 (0.1)		

	Cefuroxime		0	15345 (99.4)	4091 (99.7)	0.027	0.045	3869 (99.6)	3870 (99.7)	1	0.004
			1	96 (0.6)	13 (0.3)			14 (0.4)	13 (0.3)		
	Vitamin B1		0	15260 (98.8)	4088 (99.6)	<0.001	0.089	3868 (99.6)	3867 (99.6)	1	0.004
			1	181 (1.2)	16 (0.4)			15 (0.4)	16 (0.4)		
	Vitamin C		0	15167 (98.2)	3995 (97.3)	<0.001	0.06	3810 (98.1)	3802 (97.9)	0.569	0.015
			1	274 (1.8)	109 (2.7)			73 (1.9)	81 (2.1)		
	Potassium chloride		0	14977 (97.0)	3921 (95.5)	<0.001	0.077	3758 (96.8)	3750 (96.6)	0.658	0.011
			1	464 (3.0)	183 (4.5)			125 (3.2)	133 (3.4)		
	Sodium chloride		0	9548 (61.8)	2588 (63.1)	0.156	0.025	2526 (65.1)	2509 (64.6)	0.704	0.009
			1	5893 (38.2)	1516 (36.9)			1357 (34.9)	1374 (35.4)		
	Glucose Sodium Chloride		0	15318 (99.2)	4051 (98.7)	0.004	0.049	3853 (99.2)	3844 (99.0)	0.333	0.025
			1	123 (0.8)	53 (1.3)			30 (0.8)	39 (1.0)		
	Invert Sugar Electrolytes		0	15423 (9	4094 (99.8)	0.093	0.03	3880 (99	3877 (99.8)	0.505	0.023

				9.9)				.9)			
			1	18 (0.1)	10 (0.2)			3 (0.1)	6 (0.2)		
	Water for injection		0	15335 (99.3)	4062 (99.0)	0.035	0.037	3860 (99.4)	3857 (99.3)	0.774	0.01
			1	106 (0.7)	42 (1.0)			23 (0.6)	26 (0.7)		
	Coenzyme Q10		0	15384 (99.6)	4098 (99.9)	0.037	0.044	3876 (99.8)	3877 (99.8)	1	0.006
			1	57 (0.4)	6 (0.1)			7 (0.2)	6 (0.2)		
	Levothyroxine		0	15351 (99.4)	4094 (99.8)	0.01	0.053	3874 (99.8)	3873 (99.7)	1	0.005
			1	90 (0.6)	10 (0.2)			9 (0.2)	10 (0.3)		
	Thymopentin		0	15354 (99.4)	4063 (99.0)	0.003	0.049	3859 (99.4)	3856 (99.3)	0.779	0.01
			1	87 (0.6)	41 (1.0)			24 (0.6)	27 (0.7)		
	Loratadine		0	15393 (99.7)	4102 (100.0)	0.005	0.062	3883 (100.0)	3882 (100.0)	1	0.023
			1	48 (0.3)	2 (0.0)			0 (0.0)	1 (0.0)		
	Oxaliplatin		0	15414 (99.8)	4101 (99.9)	0.209	0.029	3880 (99.9)	3880 (99.9)	1	<0.001
			1	27 (0.2)	3 (0.1)			3 (0.1)	3 (0.1)		

	Edaravone		0	15365 (99.5)	4079 (99.4)	0.42	0.016	3858 (99.4)	3859 (99.4)	1	0.003
			1	76 (0.5)	25 (0.6)			25 (0.6)	24 (0.6)		
	Aspirin clears heat		0	14557 (94.3)	3954 (96.3)	<0.001	0.098	3743 (96.4)	3744 (96.4)	1	0.001
			1	884 (5.7)	150 (3.7)			140 (3.6)	139 (3.6)		
	Terbutaline		0	15413 (99.8)	4087 (99.6)	0.01	0.043	3873 (99.7)	3869 (99.6)	0.54	0.019
			1	28 (0.2)	17 (0.4)			10 (0.3)	14 (0.4)		
	Compound paracetamol		0	15275 (98.9)	4080 (99.4)	0.006	0.054	3859 (99.4)	3860 (99.4)	1	0.003
			1	166 (1.1)	24 (0.6)			24 (0.6)	23 (0.6)		
	Isosorbide dinitrate		0	15240 (98.7)	4045 (98.6)	0.549	0.012	3838 (98.8)	3839 (98.9)	1	0.002
			1	201 (1.3)	59 (1.4)			45 (1.2)	44 (1.1)		
	Felodipine		0	15099 (97.8)	4069 (99.1)	<0.001	0.111	3847 (99.1)	3850 (99.2)	0.809	0.008
			1	342 (2.2)	35 (0.9)			36 (0.9)	33 (0.8)		
	Nifedipine		0	13383 (86.7)	3787 (92.3)	<0.001	0.183	3572 (92.0)	3583 (92.3)	0.673	0.011

			1	2058 (13.3)	317 (7.7)			311 (8.0)	300 (7.7)		
	Enalapril		0	15102 (97.8)	4056 (98.8)	<0.001	0.08	3835 (98.8)	3838 (98.8)	0.835	0.007
			1	339 (2.2)	48 (1.2)			48 (1.2)	45 (1.2)		
	Valsartan		0	13610 (88.1)	3808 (92.8)	<0.001	0.159	3585 (92.3)	3600 (92.7)	0.546	0.015
			1	1831 (11.9)	296 (7.2)			298 (7.7)	283 (7.3)		
	Fenofibrate		0	15284 (99.0)	4092 (99.7)	<0.001	0.09	3868 (99.6)	3871 (99.7)	0.7	0.013
			1	157 (1.0)	12 (0.3)			15 (0.4)	12 (0.3)		
	Lovastatin		0	15360 (99.5)	4100 (99.9)	<0.001	0.077	3880 (99.9)	3879 (99.9)	1	0.009
			1	81 (0.5)	4 (0.1)			3 (0.1)	4 (0.1)		
	Simvastatin		0	14310 (92.7)	3946 (96.2)	<0.001	0.152	3726 (96.0)	3727 (96.0)	1	0.001
			1	1131 (7.3)	158 (3.8)			157 (4.0)	156 (4.0)		
	aspirin		0	10997 (71.2)	3433 (83.7)	<0.001	0.301	3225 (83.1)	3240 (83.4)	0.671	0.01
			1	444	671			658	643		

				4 (28.8)	(16.3)			(16.9)	(16.6)		
	Pantoprazole		0	15051 (97.5)	3936 (95.9)	<0.001	0.088	3759 (96.8)	3764 (96.9)	0.794	0.007
			1	390 (2.5)	168 (4.1)			124 (3.2)	119 (3.1)		
	Diammonium glycyrrhizinate		0	15424 (99.9)	4093 (99.7)	0.032	0.036	3878 (99.9)	3875 (99.8)	0.579	0.019
			1	17 (0.1)	11 (0.3)			5 (0.1)	8 (0.2)		
	Lactulose		0	15419 (99.9)	4092 (99.7)	0.066	0.032	3876 (99.8)	3876 (99.8)	1	<0.001
			1	22 (0.1)	12 (0.3)			7 (0.2)	7 (0.2)		
	Magnesium Isoglycyrrhizinate		0	15404 (99.8)	4081 (99.4)	0.002	0.051	3871 (99.7)	3869 (99.6)	0.844	0.009
			1	37 (0.2)	23 (0.6)			12 (0.3)	14 (0.4)		
	Torsemide		0	15361 (99.5)	4065 (99.0)	0.002	0.051	3860 (99.4)	3857 (99.3)	0.774	0.01
			1	80 (0.5)	39 (1.0)			23 (0.6)	26 (0.7)		
	Furosemide		0	15061 (97.5)	3980 (97.0)	0.05	0.034	3799 (97.8)	3789 (97.6)	0.495	0.017
			1	380 (2.5)	124 (3.0)			84 (2.2)	94 (2.4)		
	Tamsulosin		0	153	4075	0.45	0.01	385	3860	1	0.00

				50 (99.4)	(99.3)	8	5	9 (99.4)	(99.4)		3
			1	91 (0.6)	29 (0.7)			24 (0.6)	23 (0.6)		
	Hemocoagulase		0	15420 (99.9)	4088 (99.6)	0.002	0.05	3878 (99.9)	3878 (99.9)	1	<0.001
			1	21 (0.1)	16 (0.4)			5 (0.1)	5 (0.1)		
	Low molecular weight heparin calcium		0	15188 (98.4)	4011 (97.7)	0.008	0.045	3812 (98.2)	3813 (98.2)	1	0.002
			1	253 (1.6)	93 (2.3)			71 (1.8)	70 (1.8)		
	Cold and heat		0	15357 (99.5)	4098 (99.9)	0.001	0.068	3873 (99.7)	3877 (99.8)	0.453	0.023
			1	84 (0.5)	6 (0.1)			10 (0.3)	6 (0.2)		
	Cold Remedy		0	15387 (99.7)	4102 (100.0)	0.002	0.068	3882 (100.0)	3881 (99.9)	1	0.013
			1	54 (0.3)	2 (0.0)			1 (0.0)	2 (0.1)		
	Anti-inflammatory and choleretic		0	15416 (99.8)	4103 (100.0)	0.056	0.045	3883 (100.0)	3882 (100.0)	1	0.023
			1	25 (0.2)	1 (0.0)			0 (0.0)	1 (0.0)		
	Xinhuang		0	15373 (99.6)	4097 (99.8)	0.019	0.049	3879 (99.9)	3876 (99.8)	0.546	0.021
			1	68	7			4	7		

				(0.4)	(0.2)			(0.1)	(0.2)		
	Sanguisorba officinalis		0	15427 (99.9)	4092 (99.7)	0.004	0.046	3877 (99.8)	3878 (99.9)	1	0.007
			1	14 (0.1)	12 (0.3)			6 (0.2)	5 (0.1)		
	Steady Heart		0	15166 (98.2)	4060 (98.9)	0.002	0.06	3843 (99.0)	3843 (99.0)	1	<0.001
			1	275 (1.8)	44 (1.1)			40 (1.0)	40 (1.0)		
	Thirst		0	15014 (97.2)	4036 (98.3)	<0.001	0.075	3811 (98.1)	3815 (98.2)	0.798	0.008
			1	427 (2.8)	68 (1.7)			72 (1.9)	68 (1.8)		
	Ginkgo biloba Chinese medicine		0	14840 (96.1)	4018 (97.9)	<0.001	0.106	3799 (97.8)	3802 (97.9)	0.875	0.005
			1	601 (3.9)	86 (2.1)			84 (2.2)	81 (2.1)		
	Salvianolate		0	15392 (99.7)	4083 (99.5)	0.088	0.03	3865 (99.5)	3866 (99.6)	1	0.004
			1	49 (0.3)	21 (0.5)			18 (0.5)	17 (0.4)		
	Compound Danshen		0	14471 (93.7)	3975 (96.9)	<0.001	0.149	3745 (96.4)	3760 (96.8)	0.378	0.021
			1	970 (6.3)	129 (3.1)			138 (3.6)	123 (3.2)		
	Safflower yellow		0	15354	4081 (99.4)	1	<0.001	3862	3863 (99.5)	1	0.004

			(99.4)				(99.5)			
		1	87 (0.6)	23 (0.6)			21 (0.5)	20 (0.5)		
	Activate blood circulation and relieve pain	0	15394 (99.7)	4101 (99.9)	0.015	0.053	3882 (100.0)	3880 (99.9)	0.617	0.023
		1	47 (0.3)	3 (0.1)			1 (0.0)	3 (0.1)		
	Salvia miltiorrhiza	0	15135 (98.0)	4063 (99.0)	<0.001	0.081	3840 (98.9)	3843 (99.0)	0.825	0.008
		1	306 (2.0)	41 (1.0)			43 (1.1)	40 (1.0)		
	Intraocular lens implantation	0	15407 (99.8)	4083 (99.5)	0.003	0.048	3870 (99.7)	3867 (99.6)	0.71	0.013
		1	34 (0.2)	21 (0.5)			13 (0.3)	16 (0.4)		
	Vitreous surgery	0	15424 (99.9)	4095 (99.8)	0.143	0.027	3881 (99.9)	3881 (99.9)	1	<0.001
		1	17 (0.1)	9 (0.2)			2 (0.1)	2 (0.1)		
	Diagnostic procedures of the stomach	0	15403 (99.8)	4079 (99.4)	<0.001	0.056	3865 (99.5)	3862 (99.5)	0.748	0.011
		1	38 (0.2)	25 (0.6)			18 (0.5)	21 (0.5)		
	Soft tissue x-rays of the face, head, and neck	0	15381 (99.6)	4072 (99.2)	0.002	0.051	3853 (99.2)	3856 (99.3)	0.79	0.009
		1	60 (0.	32 (0.8)			30 (0.	27 (0.7)		

				4)				8)			
	Abdominal soft tissue X-ray examination		0	15413 (99.8)	4085 (99.5)	0.002	0.05	3871 (99.7)	3873 (99.7)	0.831	0.01
			1	28 (0.2)	19 (0.5)			12 (0.3)	10 (0.3)		
